# Morphological, histological and gene-expression analyses on stolonization in the Japanese Green Syllid, *Megasyllis nipponica* (Annelida, Syllidae)

**DOI:** 10.1038/s41598-023-46358-8

**Published:** 2023-11-22

**Authors:** Mayuko Nakamura, Kohei Oguchi, Daisuke S. Sato, Sumika Kato, Masanori Okanishi, Yoshinobu Hayashi, M. Teresa Aguado, Toru Miura

**Affiliations:** 1 Misaki Marine Biological Station, School of Science, The University of Tokyo, Misaki, Miura, Kanagawa 238-0225 Japan; 2 Department of Biological Sciences, Graduate School of Science, The University of Tokyo, Hongo, Bunkyo, Tokyo 113-0033 Japan; 3 Faculty of Human Environmental Studies, Hiroshima Shudo University, Ozuka-Higashi, Asaminami, Hiroshima, 731-3195 Japan; 4 Department of Biology, Keio University, Hiyoshi, Yokohama, 223-8521 Japan; 5 Animal Evolution and Biodiversity, Georg-August-Universität Göttingen, 37073 Göttingen, Germany

**Keywords:** Morphogenesis, Pattern formation, Developmental biology, Evolution, Zoology

## Abstract

Benthic annelids belonging to the family Syllidae (Annelida, Errantia, Phyllodocida) exhibit a unique reproduction mode called “schizogamy” or “stolonization”, in which the posterior body part filled with gametes detaches from the original body, as a reproductive unit (stolon) that autonomously swims and spawns. In this study, morphological and histological observations on the developmental processes during stolonization were carried out in *Megasyllis nipponica*. Results suggest that the stolon formation started with maturation of gonads, followed by the formation of a head ganglion in the anteriormost segment of the developing stolon. Then, the detailed stolon-specific structures such as stolon eyes and notochaetae were formed. Furthermore, expression profiles of genes involved in the anterior–posterior identity (Hox genes), head determination, germ-line, and hormone regulation were compared between anterior and posterior body parts during the stolonization process. The results reveal that, in the posterior body part, genes for gonadal development were up-regulated, followed by hormone-related genes and head-determination genes. Unexpectedly, Hox genes known to identify body parts along the anterior–posterior axis showed no significant temporal expression changes. These findings suggest that during stolonization, gonad development induces the head formation of a stolon, without up-regulation of anterior Hox genes.

## Introduction

Among bilaterians, the anterior–posterior body axis is widely conserved^1^, and determined early during embryogenesis, in most cases^2^. However, there are some exceptions such as fragmentation followed by regeneration, in which adult animals develop a new body axis keeping the original bilaterian symmetry^3–5^. Additionally, in several annelid species of Syllidae, an interesting developmental process occurs when animals are sexually mature, known as “stolonization” or “schizogamy”^6^. Syllidae is one of the most speciose taxa within Annelida, with more than 70 genera and nearly 1000 valid species^7,8^, and diverse reproductive modes^6,9,10^. Although most syllid species are benthic, they produce “epitokes”, i.e., sexually mature worms, which swim and spawn gametes, in the reproductive season. This pattern of reproduction is called “epitoky”^11^, which is further classified into two types, i.e., epigamy and schizogamy^6,9^. Epigamy is considered as the plesiomorphic reproductive mode^12^, in which the entire body of a benthic individual is transformed into an epitokous morph. In contrast, in schizogamy, which is also called stolonization, only a posterior part of a mature individual becomes an epitokous unit filled with gametes, termed a “stolon”, which exclusively devotes to reproduction by detaching from the original benthic individual for spawning (Supplementary Fig. 1)^6^. After stolon detachment, the original benthic individual, termed the “stock”, regenerates its posterior segments so that they can repeatedly produce stolons^6^.

Since fully developed stolons have sensory organs such as eyes and antennae, well-developed muscular systems and swimming notochaetae^13–18^, and swim in response to the presence of opposite-sex stolons^19^, they are expected to possess an independent and functional nervous system. It has also been noted in several syllid species that a brain-like nerve structure is newly formed in stolons^20,21^. Although the stolon morphology and the stolonization process have been reported in some species^11,13–18^, detailed morphological examinations by using newer techniques like immunohistochemistry and fluorescence are still required.

The species *Megasyllis nipponica* distributed all around Japan in intertidal and subtidal zones^22^, with a stable laboratory-rearing system and transcriptome data available^19,23^, has the potential to provide an excellent experimental basis for further developmental studies. In this study, morphological and histological observations were carried out to compare stolons and stocks. The developmental stages during the stolonization were defined based on morphological and histological transitions. Furthermore, expression analyses of candidate genes expected to be required for stolonization were performed and compared among developmental stages. Based on previous knowledge reported from other annelids and different animal lineages, genes known to be responsible for reproductive regulation, pattering of animal bodyplans (so-called “tool-kit genes”), and some endocrine regulators were selected. These candidates included genes for gonadal development (*piwi*, *vasa* and *nanos*)^24–26^, anterior-posterior axis information (Hox genes)^27,28^, and head-determination (*six3*, *otx*, *pax6*, *nk2.1*)^29^.

Also, since it is well known in arthropods, two major endocrine factors, i.e., juvenile hormones (sesquiterpenoids) and ecdysone, play important roles in development and reproduction^30,31^, the genes related to these factors were also investigated in this study. In addition, it has been recently found that sesquiterpenoids are implied in stolonization of one syllid species^32^. In annelids, environmental factors such as lunar and/or tidal cycles are important for controlling reproductive cycles^33,34^, so that endocrine controls are predicted to contribute to the regulation^35,36^.

## Results

### Morphological and histological comparisons between stocks and stolons

**Figure 1:**
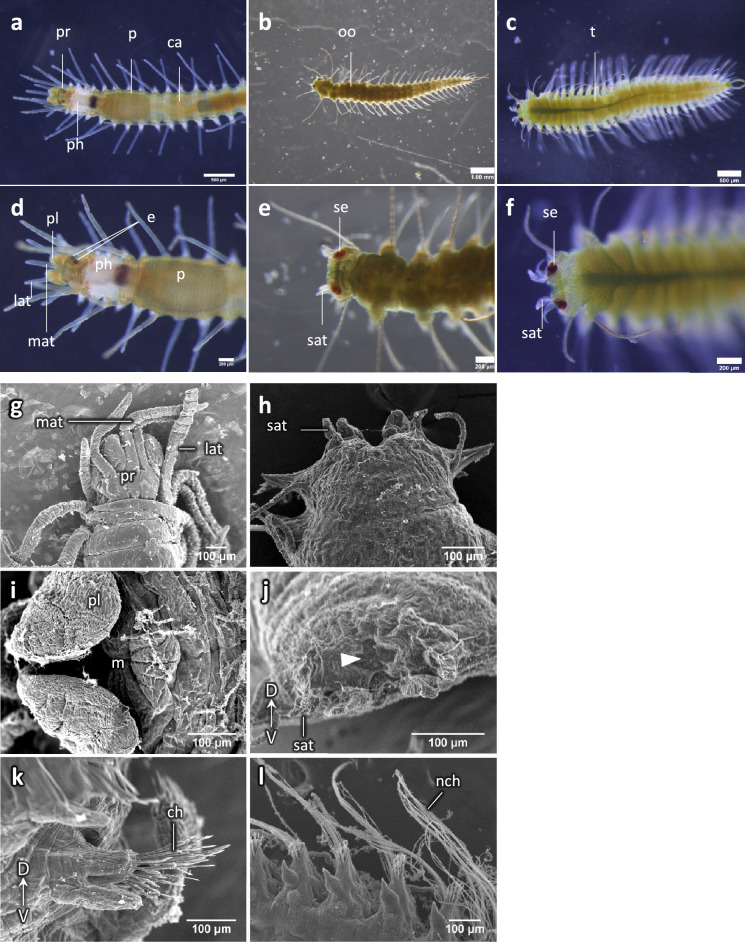
Morphological differences between stolon and stock. (**a**)–(**f**) Observations using a stereoscopic microscope; (**a**) anterior part of a stock, (**b**) female stolon, (**c**) male stolon, (**d**)–(**f**) magnified views of (**a**)–(**c**) (stock, female stolon, male stolon, respectively). (**g**)–(**l**) SEM images of stocks and stolons; Dorsal views of stock head (**g**) and an anterior part of a male stolon (**h**), (**i**) ventral view of stock mouth, (**j**) anterior end of a male stolon, (**k**) parapodia of a stock, (**l**) parapodia of a male stolon. An arrowhead shows a vestigial opening of a digestive tube. *ca* caeca, *ch* chaetae, *e* eye, *lat* lateral antenna, *m* mouth, *mat* median antenna, *nch* notochaetae, *oo* oocytes, *p* proventricle, *ph* pharynx, *pl* palps, *pr* prostomium, *sat*, stolon antenna, *se* stolon eye, *t*, testis.

Morphological observations made through the transparent body wall showed that stocks possessed a functional digestive tube morphologically differentiated into several parts, including a pharynx, a proventricle and caeca (Fig. 1a). In contrast, stolons had a undifferentiated simple digestive tube. In mature female stolons, eggs were observed through the body wall (Fig. 1b). Mature male stolons were more yellowish, and filled with sperm (testes) (Fig. 1c). Stocks had two pairs of eyes, a pair of palps, and three antennae on the head (Fig. 1d), while stolons, regardless of sex, had two pairs of eyes that were larger than those of the stock and two pairs of short antennae (Fig. 1e, f), while lacking palps (as in stolons of other syllids).

SEM observations revealed that stocks had three annulated antennae arising from the dorsal side of the prostomium (Fig. 1g), while stolons had two pairs of short antennae arising from the anteriormost segment (Fig. 1h). No difference was identified on the surface structure of eyes between the two types. In stocks, there was a functional mouth on the ventral side (Fig. 1i). In contrast, stolons did not have such a functional mouth, although a vestigial opening of the digestive tube was observed at the anterior end (Fig. 1j, arrowhead), where the stolon detached from the stock. Neurochaetae on stocks and stolons were compound (Fig. 1k); in addition, developed stolons showed long swimming simple notochaetae (Fig. 1l, nch). No structural difference between sexes was observed in the external morphology of stolons or stocks.

**Figure 2:**
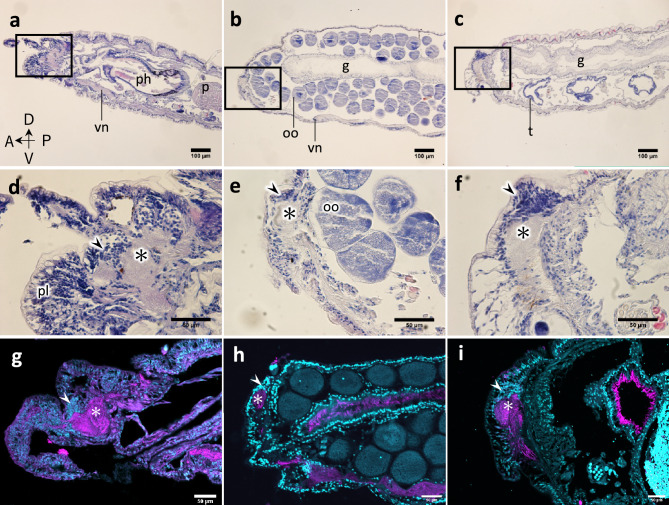
Histological comparisons between stock (**a**, **d**, **g**) and stolon (**b**, **e**, **h**: female stolon; **c**, **f**, **i**: male stolon) heads. Sagittal section of a head stained with hematoxylin and eosin (**a**)–(**f**). (**d**)–(**f**) Magnified views of rectangular regions of (**a**)–(**c**) respectively. (**g**)–(**i**) Sagittal sections stained with anti-acetylated-alpha-tubulin antibody (magenta) and DAPI (cyan). Asterisk shows nervous tissue, and arrowheads show cell-dense regions. *g* gut, *oo* oocytes, *p* proventricle, *ph* pharynx, *pl* palps, *t* testis, *vn* ventral nerve cord.

Histological observations revealed that stocks possessed a pharynx and a proventricle with well-developed muscular tissues (Fig. 2a). In contrast, in stolons, such tissues were completely absent, but a simple digestive tube was present (Fig. 2b, c). Coeloms of female stolons (Fig. 2b) were filled with oocytes, while in male stolons (Fig. 2c), pairs of testes were observed within segments. In stocks, a brain and thick neuropils were observed in the head region (Fig. 2d). In the first anterior segment of both female and male stolons, nerve tissues contained numerous neuronal somata with nuclei (Fig. 2e, f, asterisk).

To visualize the detailed nervous structure, immunohistochemistry with anti-acetylated-alpha-tubulin antibody was carried out. In the stock head, nerve tissues were observed in a dorsal region (Fig. 2g, asterisk). Adjacent to nerve tissues (neuropiles), a region was observed containing numerous nuclei considered to be neural somata (Fig. 2g, arrowhead). This part at the anteriormost segment is suggested to be a cerebral ganglion of the stock. In stolon heads (male and female), nerve tissues were also observed in a dorsal region of the anteriormost segment. As well, regions containing numerous nuclei were observed adjacent to neuropiles (Fig. 2h, i). Although the basic neural structures of the head ganglion were shared between the stock and stolons, the total volume of the ganglion was smaller in the latter. Also, no differences of neural structures were detected between female and male stolons.

### Staging of stolonization

**Figure 3:**
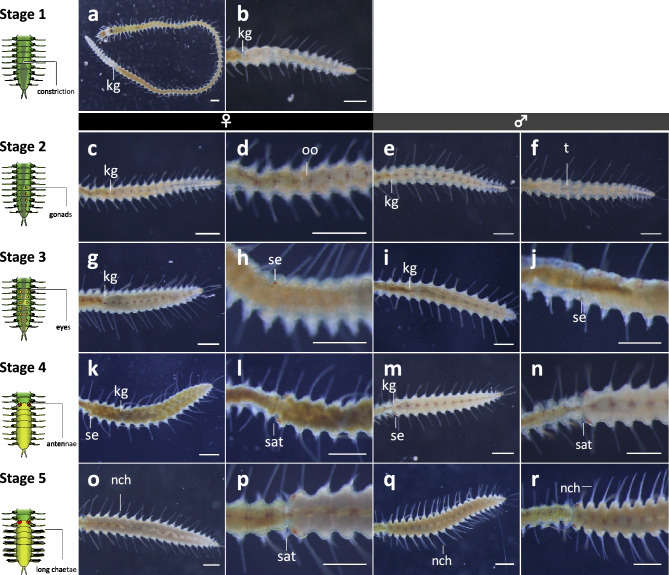
Developmental stages of stolonization process, based on images observed using a stereoscopic microscope. (**a**), (**b**) Stage 1; (**c**)–(**f**) Stage 2; (**g**)–(**j**) Stage 3; (**k**)–(**n**) Stage 4; (**o**)–(**r**) Stage 5. (**c**), (**d**), (**g**), (**h**), (**k**), (**l**), (**o**), and (**p**) are female. (**e**), (**f**), (**i**), (**j**), (**m**), (**n**), (**q**), and (**r**) are male. Scale bars indicate 500 µm. *kg* kinked structure of gut, *nch* notochaetae, *oo* oocyte, *sat* stolon antenna, *se* stolon eye, *t* testis.

Based on external morphology, 6 developmental stages were defined during the stolonization process (Fig. 3). Before the onset of stolonization (Stage 1), a kinked structure of the gut (Fig. 3a, b) was observed at a relatively posterior part of the stock, and the gut from the kinked structure to the posterior end became whitish. Later (Stage 2), in segments posterior to the kinked-gut region, oocytes (Fig. 3c, d) or testes (Fig. 3e, f) were observed at the dorsal side, indicating that the stolon sex was already determined at this stage. Then (Stage 3), two pairs of eyes were formed on a segment anterior to the kinked structure (Fig. 3g–j). At this stage, the posterior part of their body showed waving movement behavior in some individuals. After that, two pairs of antennae were formed (Stage 4, Fig. 3k–n), and eyes became enlarged. At this stage, at the ventral side of the stock, in the area where the stolon was still attached, a tiny tail bud of the stock was regenerated (Fig. 3l, n, Supplementary Fig. 2). At this time, the gut of the stock was still connected to that of the developing stolon, so that the anal opening of the newly-formed stock tail was not yet formed. Then, swimming notochaetae were elongated (Fig. 3o–r), and stolon antennae became longer (Stage 5, Fig. 3p, r). The formation of the stock-tail bud progressed on the ventral side. The stolon actively vibrated and then detached from the stock (Stage 6).

**Figure 4:**
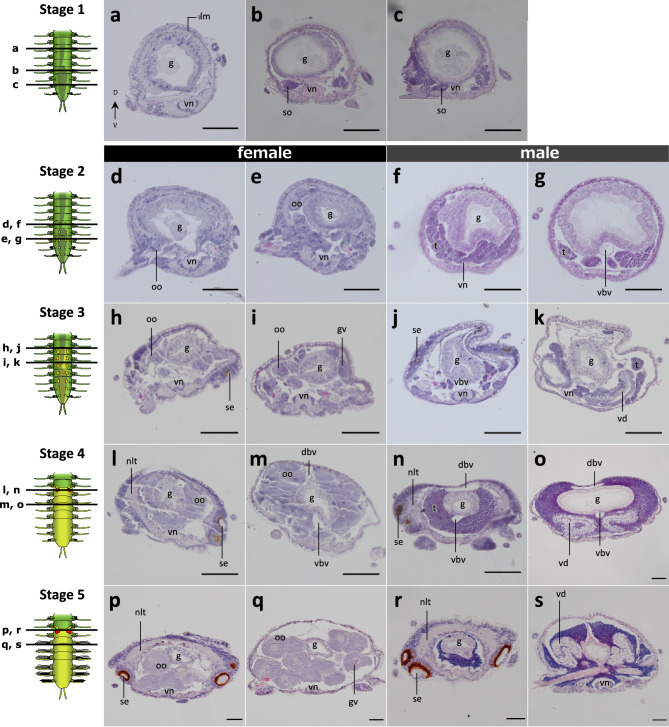
Histological transitions during stolonization. (**a**)–(**c**) Stage 1, (**d**)–(**g**) Stage 2, (**h**)–(**k**) Stage 3, (**l**)–(**o**) Stage 4, (**p**)–(**s**) Stage 5. (**d**), (**e**), (**h**), (**i**), (**l**), (**m**), (**p**), (**q**) female stolon, (**f**), (**g**), (**j**), (**k**), (**n**), (**o**), (**r**), (**s**) male stolon. The left illustrations indicate observed planes. All scale bars indicate 50 µm. *dbv* dorsal blood vessel, *g* gut, *gv* germinal vesicle, *lm* longitudinal muscle, *nlt* nerve-like tissue, *oo* oocyte, *se* stolon eye, *so* segmental organ, *t* testis, *vbv* ventral blood vessel, *vd* vas deferens, *vn* ventral nerve cord.

Histological observations were also carried out through the developmental stages defined in this study. At Stage 1, a pair of organs (identified as gonad primordia), strongly stained with hematoxylin, was observed in the ventral region of posterior segments of the stock, that later became a future stolon (Fig. 4a–c). At Stage 2, oocytes or testes were observed in coeloms of segments developing to be a female (Fig. 4d, e) or a male stolon (Fig. 4f, g). At Stage 3, dense-cell regions were observed under the body wall at both sides of the anterior end of stolons (Fig. 4h, j), forming the stolon eyes. In females, germinal vesicles were observed when gonads matured (Fig. 4i), while in males, a duct-like structure, i.e., *vas deferens*, was observed (Fig. 4k, vd). At Stage 4, nervous tissues were observed at the head region where stolon eyes were located (Fig. 4l, n), when the coelom areas were filled with oocytes or testes (Fig. 4m, o). At Stage 5, the nervous tissues were enlarged and connected to make a thick circular nerve surrounding the digestive tract (Fig. 4p, r). In female stolons, oocytes developed (Fig. 4q), while in males, sperm cells were matured and a *vas deferens* was well developed (Fig. 4s).

### Gene expression profile during stolonization

Individuals at each stage were dissected into anterior and posterior parts from which RNA was extracted for gene expression analyses by real-time quantitative PCR. Therefore, developing stolons were included in the “posterior” samples. The target candidate gene orthologs in the focal species were obtained from the transcriptome data of *M. nipponica* (Transcriptome Shotgun Assembly of DDBJ: accession numbers: ICSJ01000001–ICSJ01159970)^23^.

#### Hox genes

**Figure 5:**
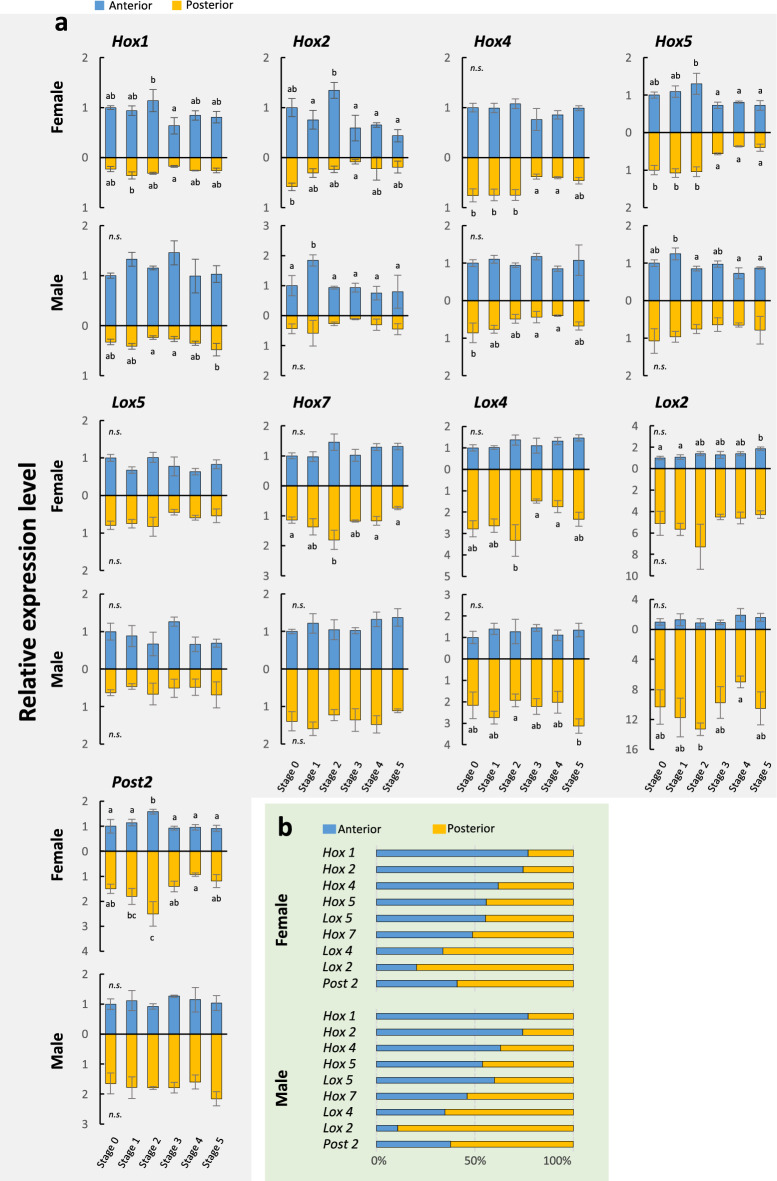
Expression patterns of 9 Hox genes (*Hox1, Hox2, Hox4, Hox5, Lox5, Hox7, Lox2, Lox4, Post2*) during stolonization, quantified by real-time qRT-PCR (**a**). Vertical axes show relative expression levels against the selected reference gene (*RP49*), and horizontal axes show stolonization stages. The blue bars represent expression in the anterior part and the orange bars represent expression in the posterior part. Different letters on bars indicate significant differences (Tukey’s test, *P* < 0.05). Error bars indicate standard deviations. (**b**) Horizontal bar charts show proportions of expression levels of the 9 Hox genes in the anterior and posterior body parts, based on the relative expression levels quantified by qPCR.

Ten Hox genes were identified, and the expression levels quantified, except *Hox3*, for which no reliable calibration curve was obtained (Fig. 5). Results showed that, for any of the Hox genes, no statistically significant up- or down-regulation was detected during stolonization (Fig. 5a). Therefore, for each Hox gene, the average expression level among all stages was calculated and the proportion of expression levels between anterior and posterior body was represented (Fig. 5b). Anterior Hox genes (*Hox1, Hox2, Hox4*) were expressed in the anterior half throughout stolonization while posterior Hox genes (*Lox4, Lox2, Post2*) were expressed posteriorly. This suggests that, in the focal species, the colinear gradient of Hox expressions along the body axis is maintained during stolonization.

#### Head-determination genes

**Figure 6:**
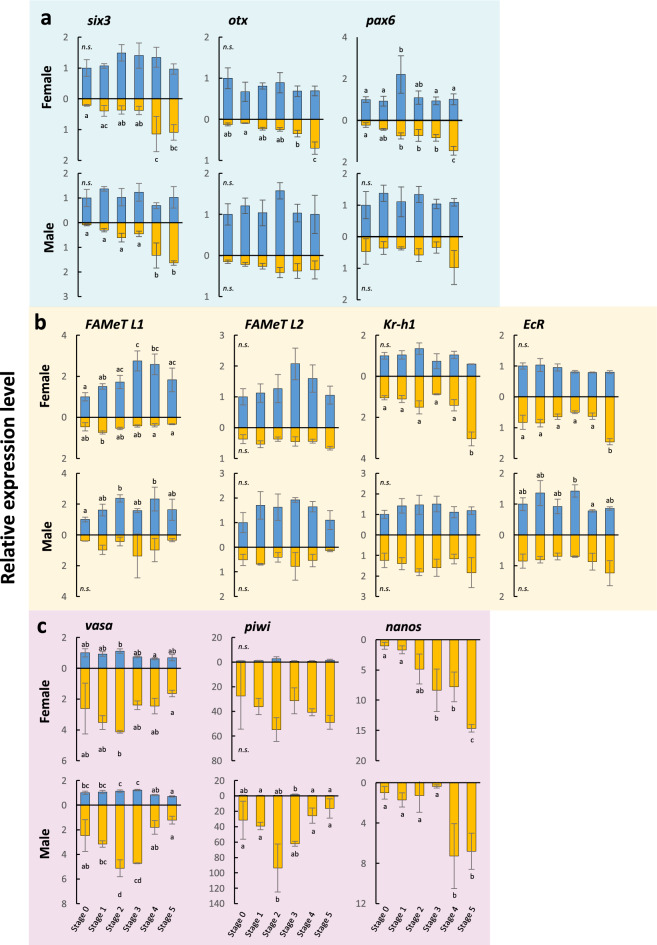
Expression patterns of head-determination, hormone-related and germ-line genes involved in the stolonization process, quantified by real-time qRT PCR. Anterior and posterior body parts are indicated by blue and orange, respectively. (**b**) Expression patterns of head-determination genes *six3*, *otx*, and *pax6*. (**c**) Expression patterns of hormone-related genes, *FAMeT* [﻿*Farnesoic acid methyl transferase*], *Kr*-*h1* [*Krüppel* *homolog 1*], and *EcR* [*Ecdysone receptor*]. (**d**) Expression patterns of germ-line genes, *vasa*, *piwi*, and *nanos.* The vertical axes show relative expression levels against the selected reference gene (*RP49*), and horizontal axes show stolonization stages. Blue and orange bars, respectively, represent anterior and posterior body parts. Different letters on bars indicate significant differences (Tukey’s test, *P* < 0.05). Error bars indicate standard deviations.

In the case of expression analyses of head-determination genes, significant upregulation during stolonization was clearly detected in the posterior half. It was shown that *six3* was upregulated in both sexes toward Stage 4, *otx* in females toward Stage 4, and *pax6* in females toward Stage 5 (Fig. 6a). In particular, *six3* was highly expressed in the posterior half in Stage 4 and Stage 5. In males, although no significant differences were detected for *otx* and *pax6* among stages, a similar tendency for *six3* was shown. The expression of *nk2.1* did not show any significant transition during the stolonization in both sexes (Supplementary Fig. 3).

#### Hormone-related genes

Two orthologs of transcripts of *FAMeT* and *JHAMT* involved in the juvenile hormone (JH) synthesis, were found in the transcriptome data of the focal species (*FAMeT L1*, *FAMeT L2* and *JHAMT L1*, *JHAMT L2*). Among these, *JHAMT* showed no significant difference among stages (Supplementary Fig. 3), while *FAMeT L1* was mainly expressed anteriorly in both sexes, showing that the expression peak was at Stage 3 in females, while from Stage 2 to Stage 4 in males (Fig. 6b). *FAMeT L2* showed a similar expression pattern to *FAMeT L1* (Fig. 6b). The downstream factors of JH and ecdysone were expressed higher in the posterior half. In particular, *Krh1* and *EcR* were upregulated toward Stage 5 in females, although there were no significant differences among stages in males. *Met* did not show significant transitions during stages in either sex (Supplementary Fig. 3).

#### Germ-line genes

In both sexes, *vasa* and *piwi* were up-regulated in the posterior half toward Stage 2, followed by a gradual decrease (Fig. 6c). The expression of *nanos* was increasing in the posterior half as the stage progressed. In the anterior half, the expression of *nanos* was not detected, probably due to very low levels under the recognition limit of qPCR. No sexual differences were detected for these genes.

## Discussion

### Stolons are individuals with specialized structures for swimming and reproduction

In this study, the detailed morphological and histological differences between stocks and stolons were described for *M. nipponica* (summarized in Supplementary Fig. 4). As expected, and in agreement with previous studies in other species^6,37^, the stolons of *M. nipponica* lack a functional digestive tube subdivided into a pharynx, proventricle and caeca, while they have a uniform digestive tube. Stolons of *M. nipponica*, as those described for other *Megasyllis* species^38^, are dicerous^7^, which means that they bear two pairs of enlarged eyes and two pairs of short antennae (Fig. 1). Regarding inner structures, stolons possess a ganglion, dorsally located within the anterior-most segment, which resembles the stock brain (Fig. 2). This suggests that stolons have their own central nervous system independent from the stock. The enlarged eyes of stolons appear to be functional in photoreception, and the short antennae are suggested to be used for the reception of pheromones from opposite-sex stolons. The anterior nervous structures in the head of stolons were simpler than those in stocks, which probably imply less perceptional information, as described for other species^18,21,31^. In contrast to stocks, stolons lack some additional sensory organs such as palps and ciliated nuchal organs that directly project from the brain^39,40^, which might be due to the adaptation to an immediate swimming and spawning behavior, followed by death in many cases.

### Gonad development precedes stolon head formation

**Figure 7:**
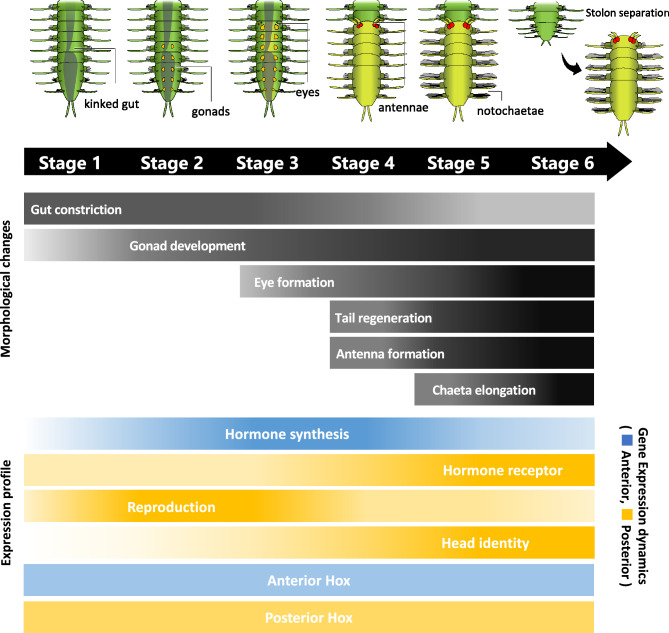
Schematic diagram showing the developmental process of stolonization. The top illustration shows staging based on morphological characteristics. The gray bands show morphological changes during developmental events. The lower bands show the transitions in gene expressions upregulated in anterior (blue) and posterior (orange) body parts.

Along the defined developmental stages during stolonization, expression profiles of candidate genes were investigated (Fig. 7). Histological observations during development revealed that, at Stage 1, cell clusters stained with hematoxylin were found on both lateral sides of segments posterior to the kinked gut (Fig. 4). Since many annelids have a pair of gonad primordia abutting on a septum^41,42^, the cell clusters observed at Stage 1 (Fig. 4b, c) are thought to be the reproductive primordia, indicating that the gonad development seems to start at this stage. At Stage 2, developing oocytes and testes were found respectively in females and males (Fig. 4d–g), indicating that the stolon sex was determined before this stage. At Stage 3, stolon eyes were formed at the head. Nerve tissues were observed interior to the stolon eyes at Stage 4, and then, at Stage 5, they enlarged at the dorsal side surrounding the gut (Fig. 4h–s), suggesting that the onset of nervous modifications follows the gonadal development. Although previous studies reported some morphological transitions during stolonization^11,16,43,44^, the detailed staging including both outer and inner morphologies had not been described in detail. However, those previous studies showed diversity of stolonization processes among species. Therefore, further comparative analyses across syllid species should be required to conclude about the evolution of stolonization processes among the syllid lineages.

Expression levels of germ-cell markers showed clear differences between anterior and posterior body regions (Fig. 6c). The expressions of *vasa* and *piwi* were up-regulated in the posterior half toward Stage 2 and then gradually decreased, while the *nanos* expression increased toward Stage 4 and 5. The expression patterns of *vasa* and *piwi* are reminiscent of those in *Capitella teleta*, where *vasa*, *nanos* and *piwi* were up-regulated in immature oocytes, whereas mature oocytes showed no expression of these^45,46^. These are also consistent with the lack of expression of germ-line markers in mature females of *Typosyllis antoni*^26^. No significant expression differences were detected between sexes, suggesting that the two sexes share similar patterns in early gametogenesis. Generally, in annelids, these three genes are also expressed in the Posterior Growth Zone (PGZ) and are known to be involved in posterior growth^47–50^. In *M. nipponica*, tail regeneration of the stock occurs at Stages 4 and 5, when *nanos* is highly expressed. Therefore, in contrast to *vasa* and *piwi*, the late expression of *nanos* could be involved in the later process of gametogenesis, or the PGZ formation of the regenerating stock tail.

Following the *vasa* and *piwi* expressions, the head-determination genes, i.e., *six3*, *otx* and *pax6*, were up-regulated, simultaneously with the tissue changes in the nervous system alterations during stolonization (Fig. 7). It could be possible that the gonad development leads the expression of head-determination genes in the stolon anteriormost segment, resulting in neuronal modifications.

### Stolon lacks body-trunk identity

Annelids exhibit metamerism, wherein the internal and external morphological features are repeated within each body segment^51^. In stolonization, however, gonads mature only in the posterior segments of the stock, which develop into a stolon, and the modification of the nervous system occurs in the first stolon segment. Therefore, it was suggested that positional information should be required to identify at least the first and the subsequent segments of the stolon. Bilaterians generally share a similar body plan along the anteroposterior axis that is determined by some specific conserved genes such as Hox genes and head-determination genes (*six3*, *otx*, *pax6*, *nk2.1*). Therefore, it was predicted that the stolonization depends on the ectopic expression of these genes. If the stolon has a similar anterior–posterior axis to that of the stock, these genes could be ectopically expressed at the region where the stolon develops.

In fact, some head-determination genes (*six3*, *otx*, *pax6*) were up-regulated in the posterior half during Stages 4 and 5 (Fig. 6a), when neural tissue develops at the anterior end of a stolon, suggesting that these genes provide the anterior identity of the stolon head. On the other hand, the expression of the anterior Hox genes (*Hox1, Hox2, Hox4*) was highly localized in the anterior half of stocks, while posterior Hox genes (*Lox4, Lox2, Post2*) were more highly expressed in the posterior region (Fig. 5), as shown in other annelids^52–54^. Furthermore, temporal changes of the expression patterns of these Hox genes were not seen during stolonization. These results may suggest that, unexpectedly, although the detailed localization patterns should be revealed by in situ hybridization in future studies, the body-trunk identities specified by Hox genes are not altered during stolon formation, and the head region is determined in the middle of the existing AP axis of the body. It is possible that the head determination genes might be co-opted downstream of positional genes such as Hox that determine the middle region, for the formation of the stolon head. The results are also consistent with the fact that stolons lack the differentiated digestive-tract components such as pharynx, proventricle, and caeca, seen in the anterior part of the stock body, and that stolons have repeated uniform body segments except for the anteriormost segment and the pygidium (Fig. 2).

### Hormonal control of stolonization

It is generally known that juvenile hormone (JH) and ecdysone are involved in molting and metamorphosis in arthropods^30,31^, in addition, these factors are also responsible for reproduction in many cases^55^. Therefore, we hypothesized that they may also be involved in stolonization, so that the expression patterns of genes involved in the up- and downstream of these pathways were also investigated in this study (Fig. 6b). As the results, *FAMeT*, one of the juvenile hormone synthases, was mainly expressed in the anterior half of the body, peaked at Stage 3, and decreased thereafter, suggesting that the JH pathway is involved in stolonization. As for the downstream factors of JH, while males did not show significant expression changes, in females, *Krh1* was gradually up-regulated toward Stage 5, following the *FAMeT* peak. This epistatic relationship is similar to that of insects^30,31^.

In *Platynereis dumerilli*, which exhibits epitoky, MF, a JH precursor is suggested to promote posterior growth while inhibiting yolk formation^34^. In females at Stage 5 in *M. nipponica*, when *Krh1* expression is maximal, the oocytes are fully mature and the stock tail is regenerating, suggesting that the JH pathway may cause an energy shift from yolk formation to development of detailed stolon morphologies. This result is consistent with the fact that MF inhibits yolk formation^36^, although it contradicts a previous finding that the expression of a downstream gene of MF (MTr) is up-regulated in individuals with mature stolons^32^. Since the expression of ecdysone receptors was also up-regulated at Stage 5, it is possible that they are involved in morphogenesis such as chaetae development, as in insect metamorphosis.

Overall, JH and ecdysone may be involved in the progression of stolon formation, although they may not act as triggers of stolon formation, because their expressions changed in the later stages of stolon formation. Further studies on other hormones which are hypothesized to contribute to sexual maturation in annelids^56,57^, should be required to comprehensively understand the endocrine mechanism underlying stolonization.

### Evolutionary implications

Based on the results obtained in this study, it is suggested that, in the evolutionary process acquiring stolonization in syllids, the basic developmental program for bilateral symmetry along the antero-posterior axis has been modified, by recruiting cephalic genes (i.e., head determination genes) at the relatively posterior body trunk, probably through some physiological factors. Presumably, it could be considered that some preexisting developmental characteristics of the ancestral worms, like the functional transition into reproductive swimming form and/or the high ability of regeneration, could be preadaptive leading to the acquisition of stolonization.

## Materials and methods

### Field collection and syllid rearing

Individuals of *Megasyllis nipponica* were collected on intertidal and subtidal zones at Oshoro Bay, Otaru, Hokkaido, or at Arai-hama Beach, Miura, Kanagawa, Japan. Red or calcareous algae belonging to Rhodophyta were collected from shallow rocky reefs, then washed in plastic trays, where syllid individuals were released. Laboratory culture of the collected individuals was carried out according to a previous study^19^. Species identification was carried out according to previous studies^22,38^. To confirm genetic differences among strains, mitochondorial cytochrome oxidase I (COI) gene were sequenced (DDBJ accession numbers: OR381444-OR381456, Supplementary Table 1). The molecular phylogenetic analysis showed that these two strains belong to the same species because the samples from two localities were included in a monophyletic clade together with the Manazuru strain whose sequence was deposited in the NCBI database (Supplementary Fig. 5).

### Morphological observations

Morphological observations on stocks and stolons during stolonization were carried out using a stereoscopic microscope (SZX-16, Olympus, Tokyo, Japan) equipped with a CCD camera (DP74, Olympus). Specimens in filtered sea water were anesthetized by menthol or 7% MgCl_2_ (w/v in distilled water) solution.

Scanning electron microscopy (SEM) was conducted for detailed morphological examinations. After anesthesia, an anterior part of a stock or a stolon was dissected and fixed in 4% (w/v) paraformaldehyde (PFA) in phosphate-buffered saline (PBS). The samples were then transferred into increasing concentrations of ethanol followed by *t*-butanol^58^. After that, they were freeze-dried using a Freeze Dryer ES-2030 (Hitachi Global, Tokyo, Japan), and coated with gold ions with an Ion Sputter E-1010 (Hitachi Global, Tokyo, Japan). Samples were observed with a scanning electron microscope JSM-5510LV (JEOL Ltd., Tokyo, Japan). At least 3 individuals were examined.

### Histological observations on paraffin sections

For histological observations of the stolonization process, paraffin sections were made as described^59^. Briefly, individuals were dissected at the region around the boundary between a stock and a stolon, and samples were fixed in 4% PFA and preserved in 70% (v/v) ethanol. Samples were dehydrated in increasing concentrations of ethanol, transferred into xylene and then embedded in paraffin. Transverse or sagittal serial Sects. (7-µm thick) were processed with a Spencer Lens microtome (Buffalo, USA) and stained with hematoxylin and eosin. Tissues on slides were observed using a BX51 Microscope equipped with a DP74 camera (Olympus, Tokyo). More than 3 individuals were examined for this process.

### Observations on central nervous system

To observe detailed nervous structures of stocks and stolons, immunological staining was performed using a monoclonal antibody against acetylated α-tubulin, that visualizes cytoskeletal microtubules, including neural axons. Samples were double-stained with DAPI (4′,6-diamidino-2-phenylindole) to examine cell density. Paraffin sections were deparaffinized, hydrated, washed in PBT (0.3% v/v Triton X-100 in PBS), and transferred to BBT (0.2% bovine serum albumin in PBT), then to NGS/BBT (2% v/v normal goat serum in BBT) for 30 min for blocking. Samples were incubated overnight at 4 °C with the primary antibody (monoclonal Mouse anti-acetylated-alpha-tubulin; MilliporeSigma, Missouri, USA) in NGS/BBT (1:500 dilution). After that, they were washed in BBT. Blocking processes were again carried out and then the samples were incubated with fluorescent-conjugated secondary antibodies (Alexa Flour 488 Goat anti-Mouse IgG, Invitrogen, Eugene, OR) in BBT (1:500 dilution ratio). After washing, the samples were stained with DAPI (5 µg/mL PBT), washed with PBT, and mounted with VECTOR Shield (Vector Laboratories, Inc., Burlingame, CA). Mounted samples were observed under a confocal laser scanning microscopy (FV3000, OLYMPUS, Tokyo). More than 3 individuals were examined for this process. Images were analyzed using FIJI software^60^.

### Gene identification

For gene-expression analyses, orthologs of candidate genes were searched in the transcriptome database of *M. nipponica* (Transcriptome Shotgun Assembly of DDBJ: accession numbers: ICSJ01000001–ICSJ01159970)^23^. The protein sequences of these candidate genes in *Platynereis dumerilli* were used as queries for tBLASTn searches against the *M. nipponica* dataset (Supplementary Tables 2–5). As the result, 5 hormone-related genes (*FAMeT* [﻿*Farnesoic acid methyl transferase*], *JHAMT* [﻿*Juvenile hormone acid O- methyltransferase*], *MeT* [*Methoprene*-*Tolerant*], *Kr*-*h1* [*Krüppel* *homolog 1*], and *EcR* [*Ecdysone Receptor*]), 3 germ-line genes (*vasa*, *piwi*, and *nanos*), 10 Hox genes (*Hox1, Hox2, Hox3, Hox4, Hox5, Lox5, Hox7, Lox2, Lox4, Post2*), and 4 head-determination genes (*six3*, *otx*, *pax6*, *nk2.1*) were identified. The obtained amino-acid sequences in *M. nipponica* were aligned with orthologous sequences in other bilaterian lineages (Supplementary Table 2–5) with AliView v.1.2.6^61^. Phylogenetic analyses using the maximum likelihood method (ML) were performed with IQ-TREE v.2.1.2^62^ to verify the orthology of candidate genes (Supplementary Figs. 6–9). The best-fitting evolutionary models were determined according to BIC (Bayesian Information Criterion), using ModelFinder^63^ implemented in IQ-TREE. ﻿Bootstrap support values were estimated using 1,000 runs.

### RNA extraction and real-time qRT-PCR

To investigate expression patterns during stolonization, 5 individuals of each sex at each developmental stage (Fig. 3) were dissected into anterior and posterior parts (more anterior to the detachment point). The anterior part included the prostomium, peristomium, and differentiated digestive tracts, while the posterior part included the region destined to be a stolon. Total RNA was extracted using ISOGEN (NIPPON GENE, Tokyo, Japan), and treated with DNaseI (Thermo Fisher Scientific, Waltham, MA, USA).

For each sample, 500 ng of total RNA was reverse transcribed with a High-Capacity cDNA Reverse Transcription Kit (Applied Biosystems, Foster City, CA, USA). Realtime quantitative PCR was then performed using SYBR Green Master Mix with the sequence detection system ABI PRISM 7500 (Applied Biosystems, Foster City, CA, USA). Primers for the realtime qRT-PCR were designed using Primer3 software^64^ based on the obtained sequences (Supplementary Table 6).

Evaluations of the appropriate candidate reference gene for quantification were carried out with the software geNorm^65^ and Normfinder^66^ among 5 candidate genes (*GAPDH* [*glyceraldehyde 3-phosphate dehydrogenase*], *18S rRNA*, *RPS9* [*Ribosomal Protein S9*] *RP49* [*ribosomal protein 49*], *EF1a* [*elongation factor 1 alpha*], and the results showed that *RP49* was the best reference gene. Data acquisition and analyses were handled with ABI Prism 7500 software ver. 2.0.4 (Applied Biosystems, Foster City, CA, USA), with the relative standard curve method. The expression level of *Hox3* was not detected since no reliable calibration curve was obtained. Relative transcript levels against the selected reference gene were visualized. For statistics, Tukey’s multiple comparisons test (*P* < 0.05) was performed after one-way ANOVA (*P* < 0.05), using R 4.0.2 (https://www.r-project.org). Three biological replicates and two technical replicates were examined for each sample category.

### Supplementary Information

Supplementary Information 1.

Supplementary Information 2.

Supplementary Information 3.

Supplementary Information 4.

Supplementary Information 5.

Supplementary Information 6.

Supplementary Information 7.

## Data Availability

The datasets generated and/or analyzed during the current study are available in the NCBI (National Center for Biotechnology Information) repository under the accession numbers OR381444-OR381456 (COI sequences for the species identification) and ICSJ00000000 (Transcriptome Shotgun Assembly for ortholog searches for the gene expression analyses).
