# Supplementary material for: Morphological, histological and gene-expression analyses on stolonization in the Japanese Green Syllid, Megasyllis nipponica (Annelida, Syllidae)
Source: Sci Rep. 2023 Nov 22;13:19419. doi: 10.1038/s41598-023-46358-8 (PMC10665476; doi:10.1038/s41598-023-46358-8)
Supplement: Supplementary file 1 — Supplementary Information 1. [file 41598_2023_46358_MOESM1_ESM.pdf]

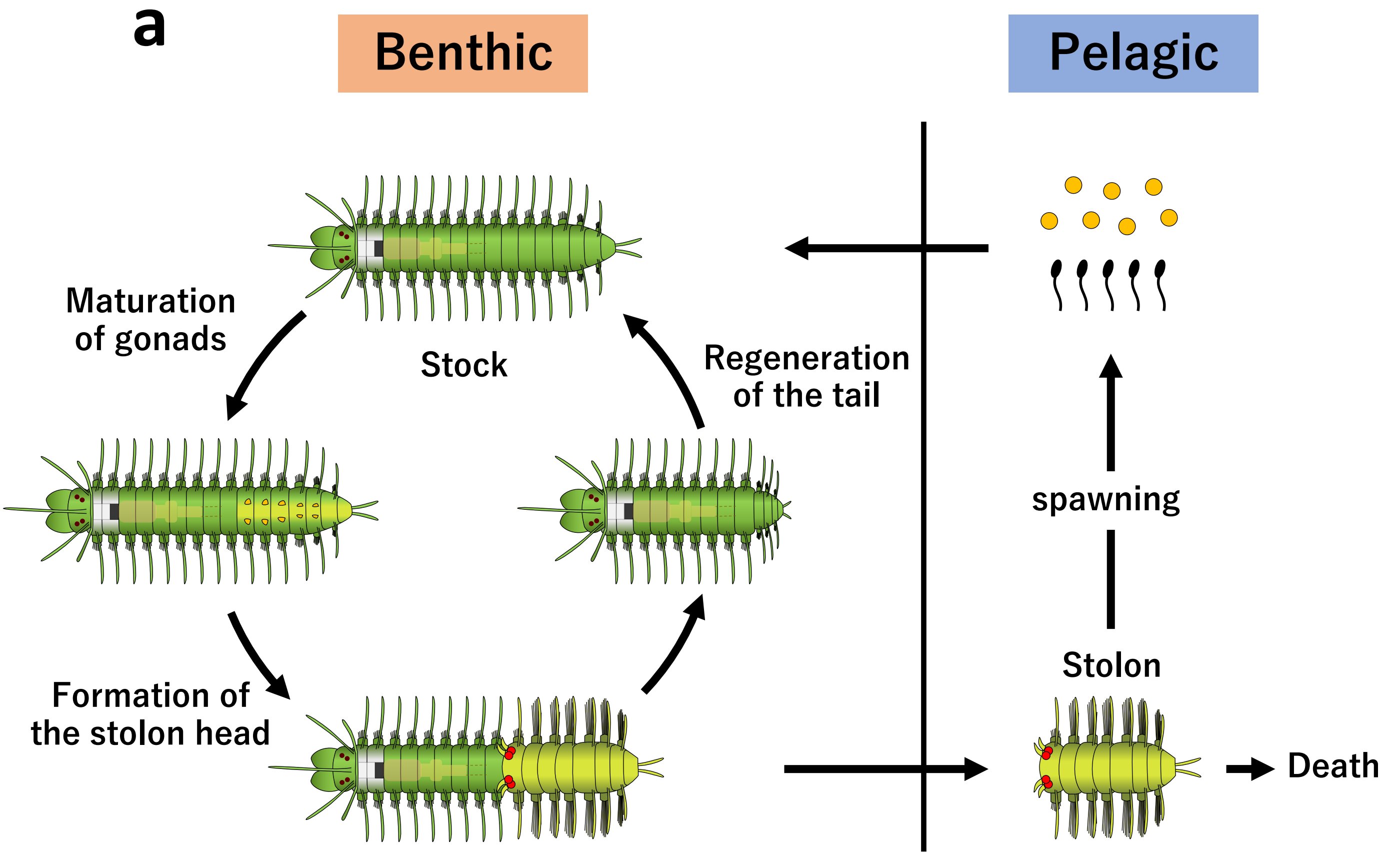

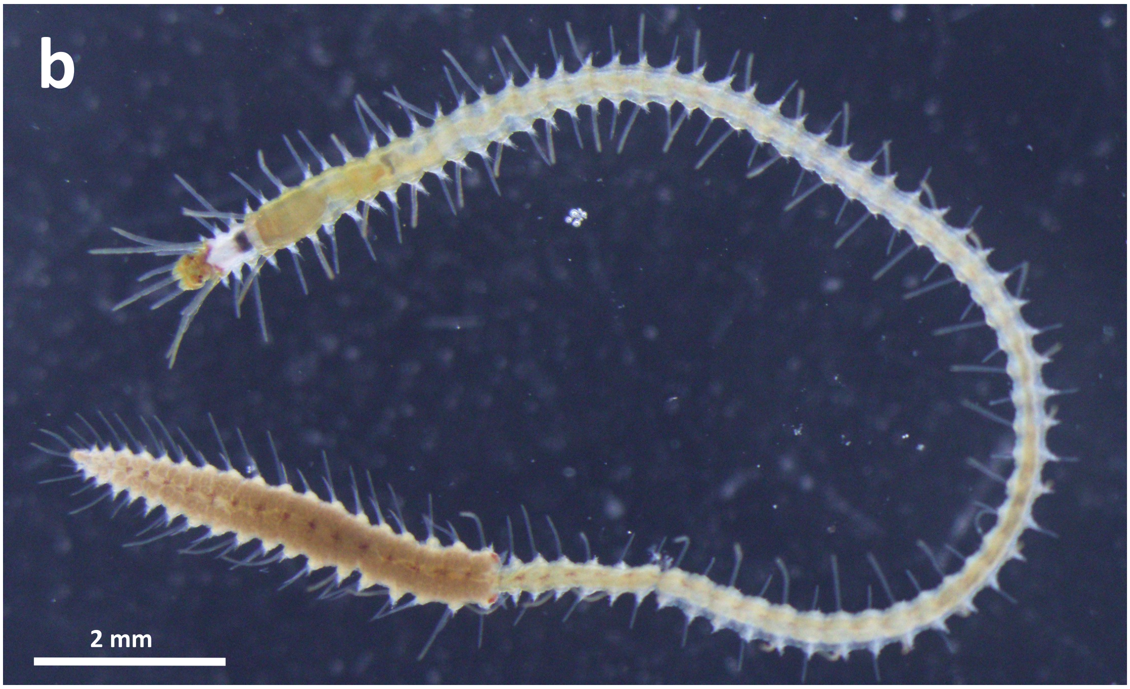

Supplementary Figure 1.

The study material in this study, *Megasyllis nipponica*. (a) Life cycle of *Megasyllis nipponica*, modified after Franke (1999) and Miura et al. (2019). (b) A mature individual of the focal species, with a developing female stolon.

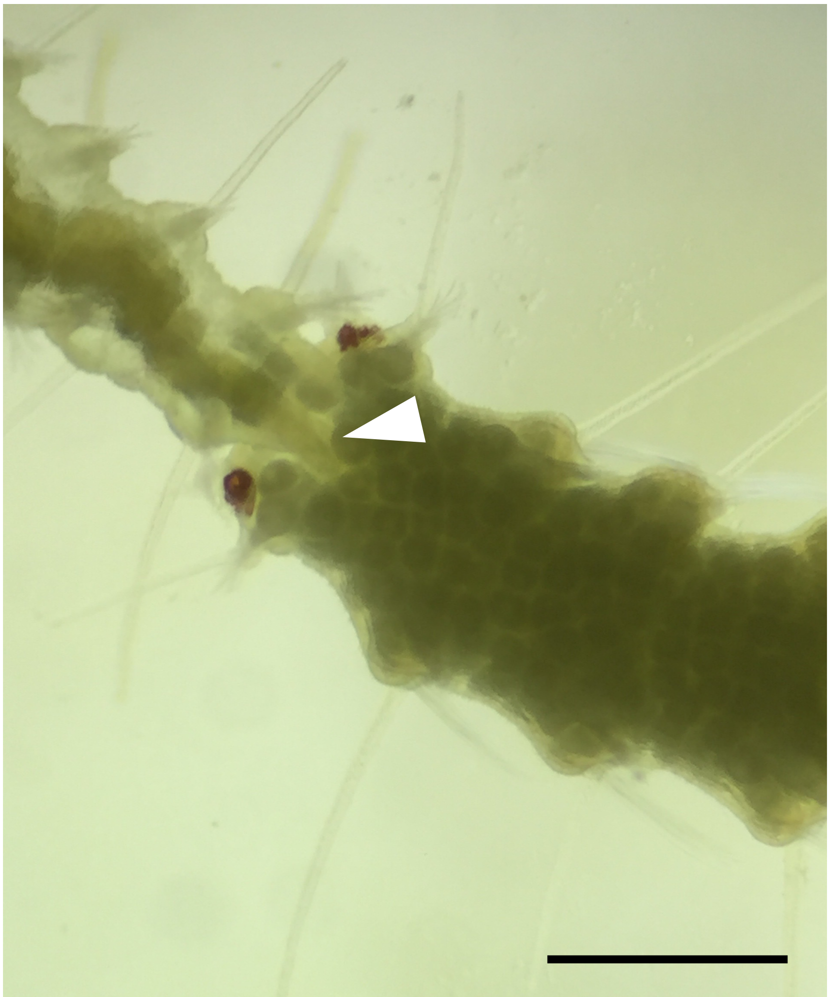

Supplementary Figure 2.

Ventral view of the detachment point between stock and stolon (Stage 5). A newly formed tail of a stock is already formed before detachment. Bar indicates 500 µm.

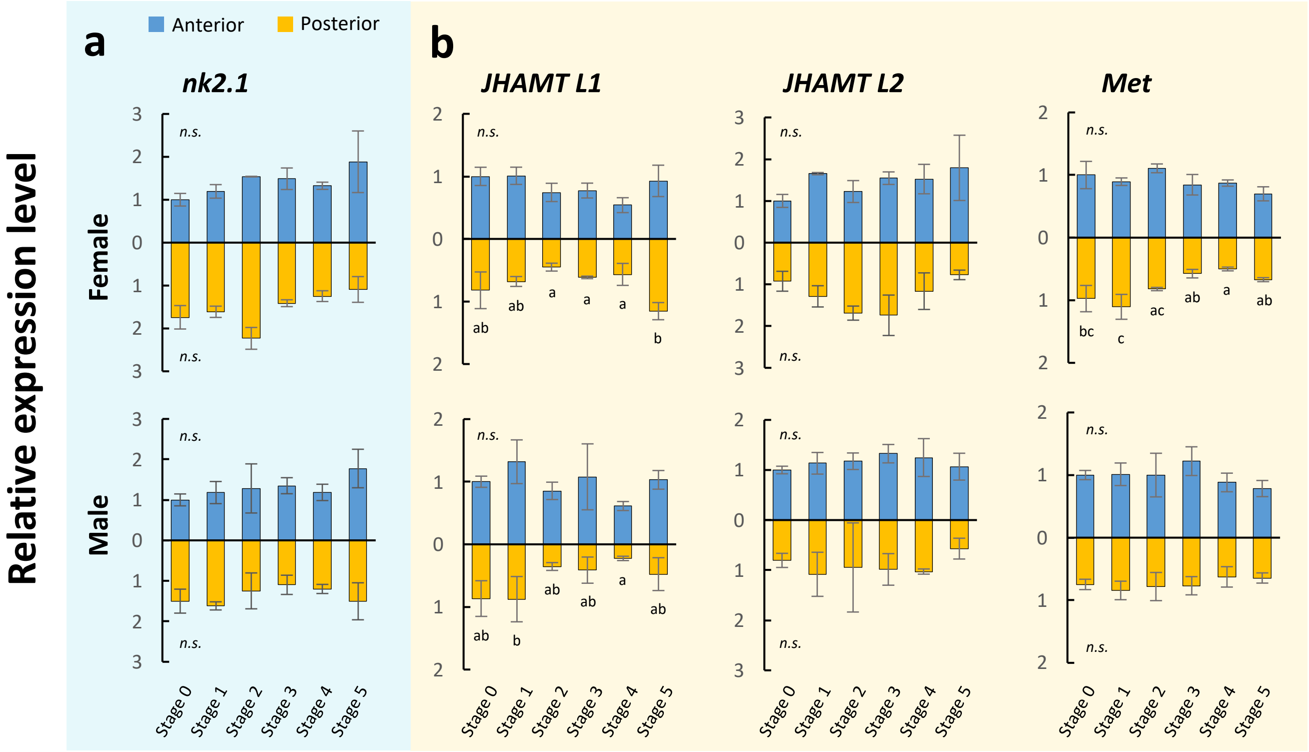

Supplementary Figure 3.

Expression patterns of candidate genes which may be related to the stolonization process, quantified by real-time qRT-PCR. (**a**) Expression patterns of a head-identification gene, *nk2.1*. (**b**) Expression patterns of hormone-related genes, *JHAMT* [*Juvenile hormone acid O-methyltransferase*] and *Met* [*Methoprene-tolerant*]. The vertical axis shows relative expression level, and the horizontal axis shows stage of stolonization. The blue bars represent expression in the anterior part and the orange bars represent expression in the posterior part. Different letters on bars indicate significant differences (Tukey's test, $P < 0.05$). The black lines indicate standard deviations.

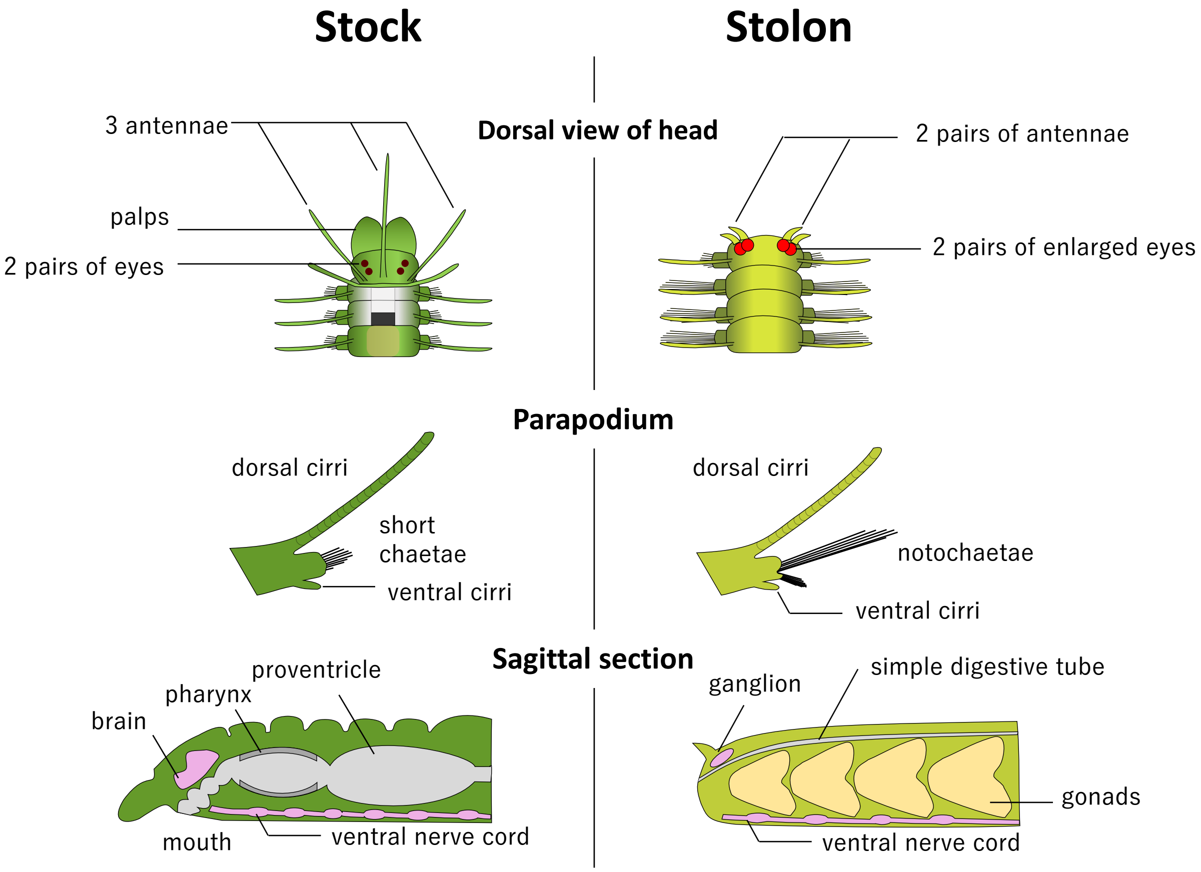

Supplementary Figure 4.
Schematic diagram showing the morphological and anatomical differences between stock and stolon.

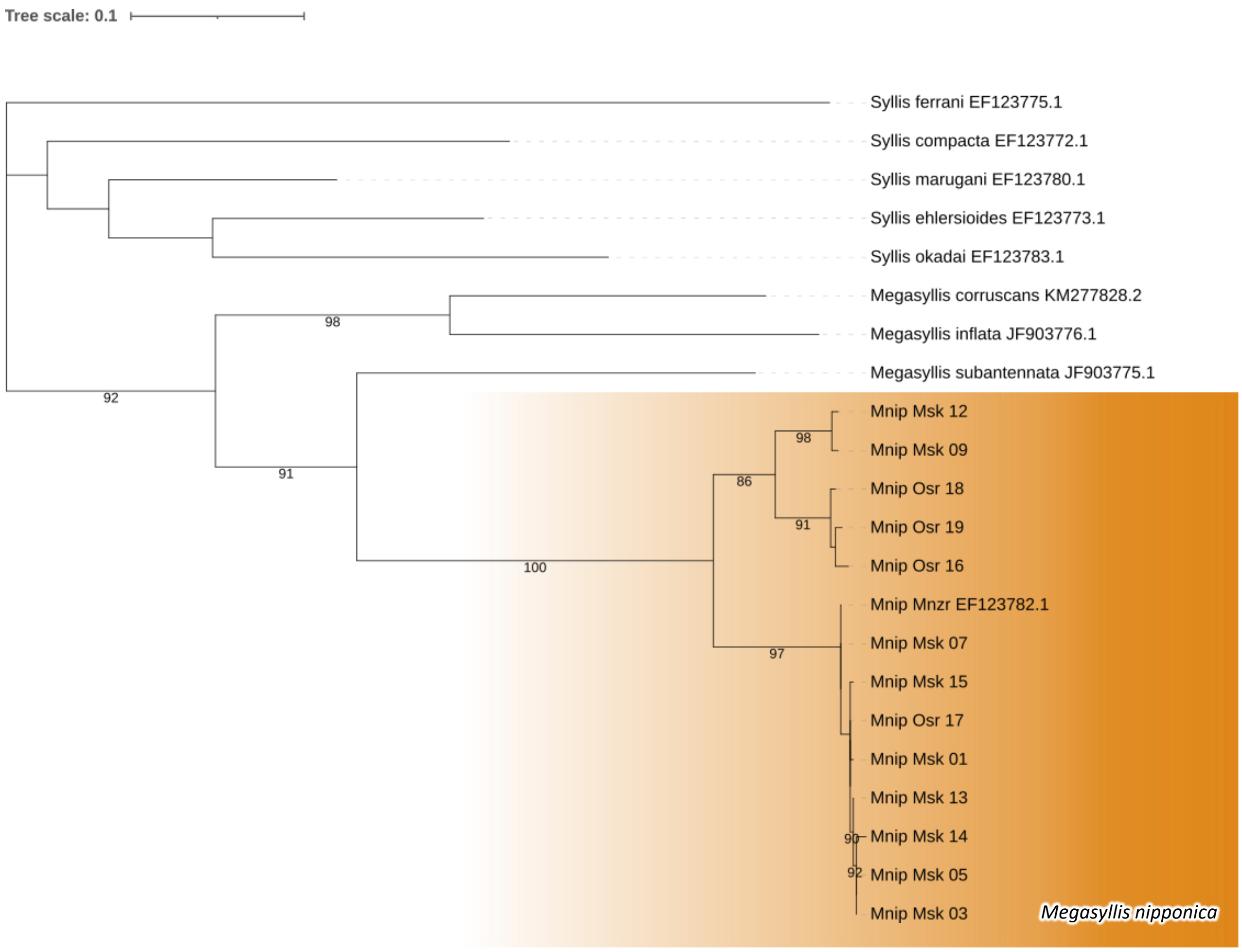

Supplementary Figure 5.

Molecular phylogenetic tree of *Megasyllis nippinica* obtained with amino-acid sequences of COI by maximum likelihood estimation. As outgroups, closely related species (*Syllis ferrani*, *Syllis compacta*, *Syllis marugani*, *Syllis ehlersiodes*, *Syllis okadai*, *Megasyllis corruscans*, *Megasyllis inflata*, *Megasyllis subantennata*) were used. TIM2+F+I+G4 was chosen as the best-fitting evolutionary model. The clade of *M. nipponica* is marked in orange. Only bootstrap values (1000 replications) larger than 80 supporting the monophyly are shown on the branches. OTU followed by the INSD accession numbers.

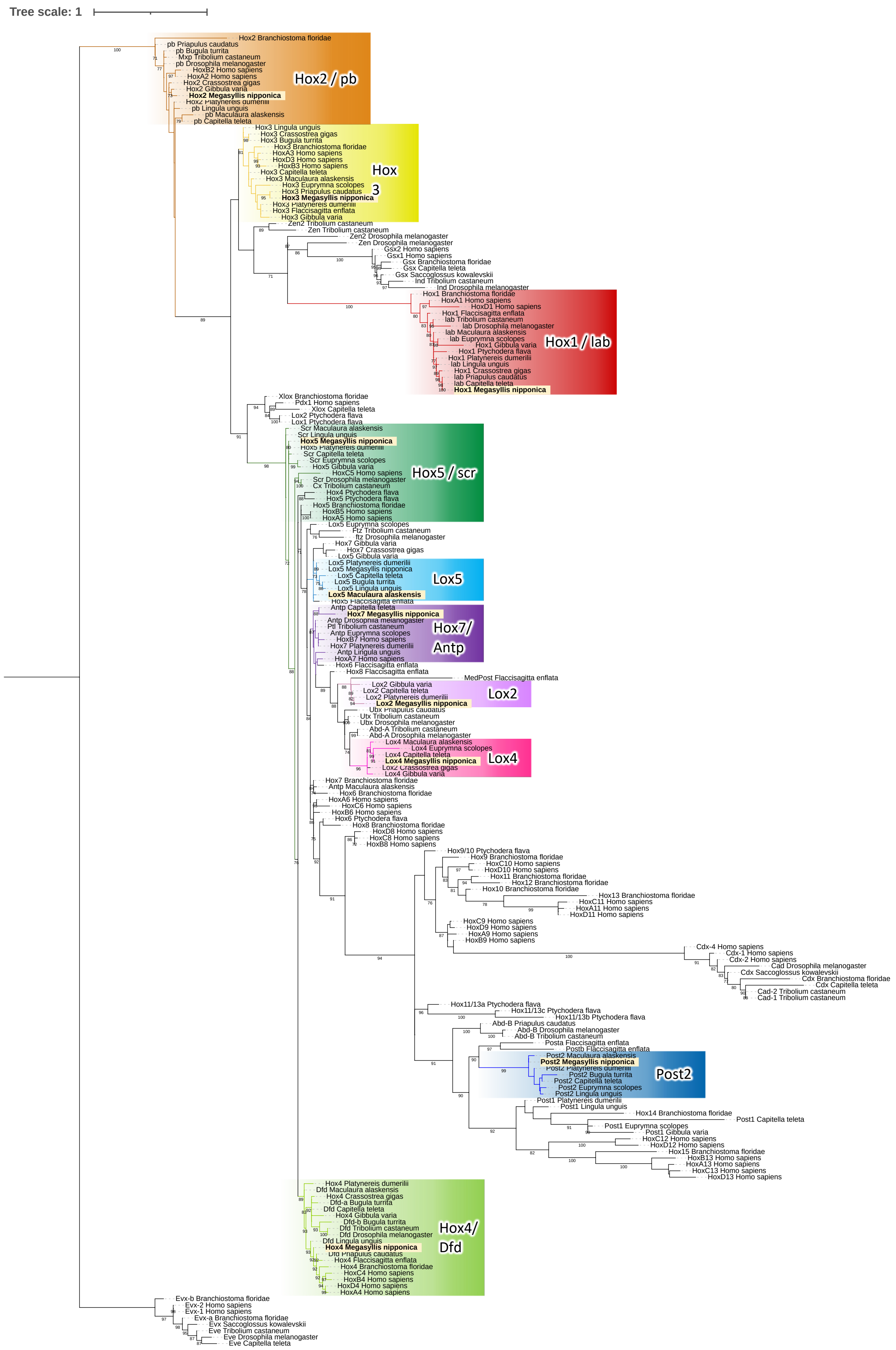

Supplementary Figure 6.

Maximum likelihood phylogenetic tree of bilaterian Hox and ParaHox genes, using the *even-skipped* (*EVX*) subfamily as outgroup. Colored boxes indicate Hox ortholog groups present in spiralian representatives. The sequences were aligned and trimmed to include the 60 amino acids of the homeodomain. LG + I + G4 was chosen as the best-fitting evolutionary model. Sequences of *M. nipponica* are highlighted by yellow. Only bootstrap values larger than 70 are shown at the nodes.

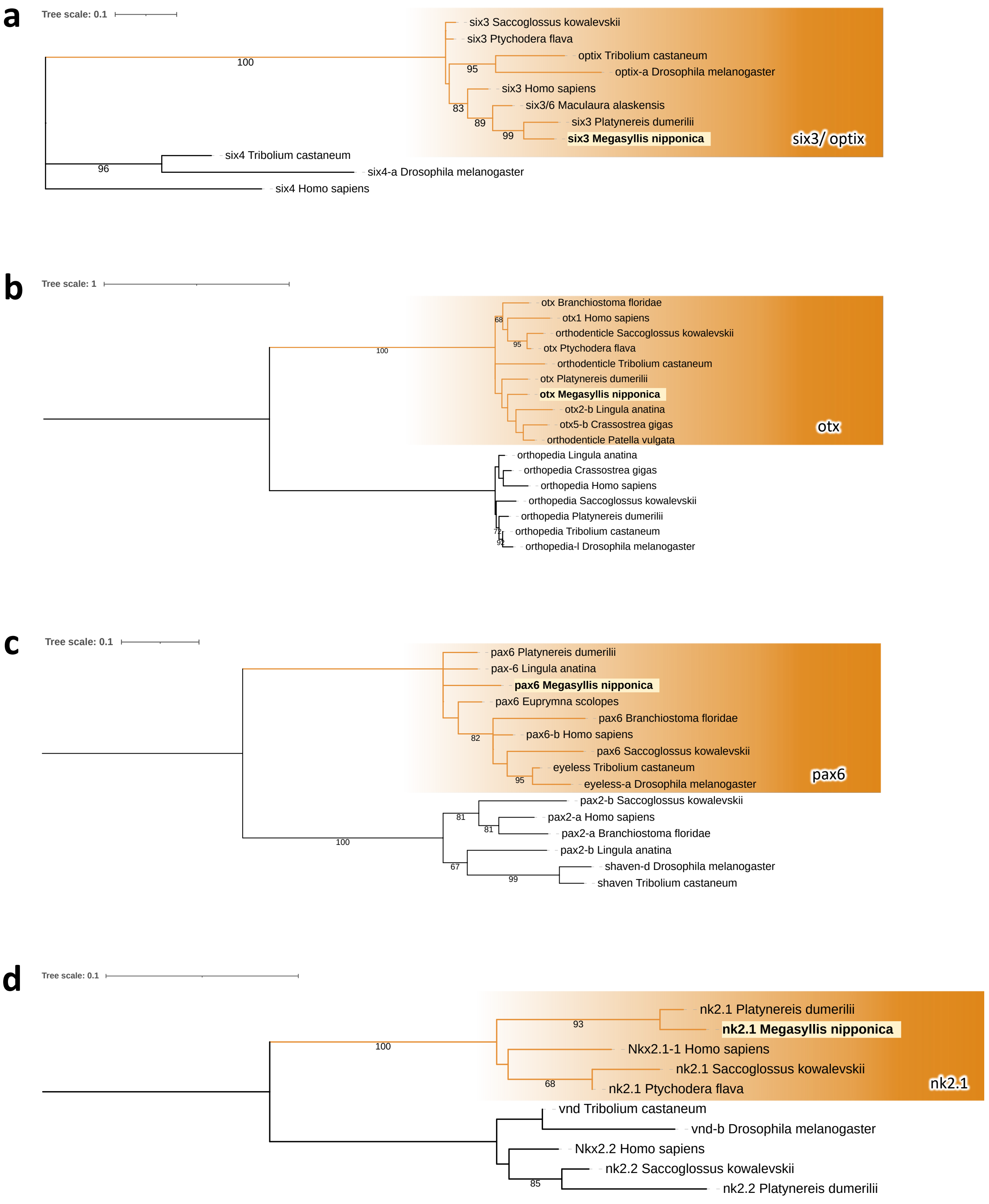

Supplementary Figure 7.

Molecular phylogenetic trees of head-identification genes. Maximum likelihood phylogenetic analyses of bilaterian *six3* (**a**), *otx* (**b**), *pax6* (**c**), and *nk2.1* (**d**), using as outgroup *six4*, *orthopedia*, *pax2*, and *nk2.2* respectively. Colored boxes indicate ortholog groups. The sequences were aligned and trimmed to include conserved region. As the best-fitting evolutionary model, (**a**) rtREV+G4, (**b**) DCMut+G4, (**c**) rtREV+R3, and (**d**) LG+G4 were chosen. *M. nipponica* sequences are highlighted by yellow. Only bootstrap values of at least 60 at nodes that supposedly represent distinct orthology groups are shown.

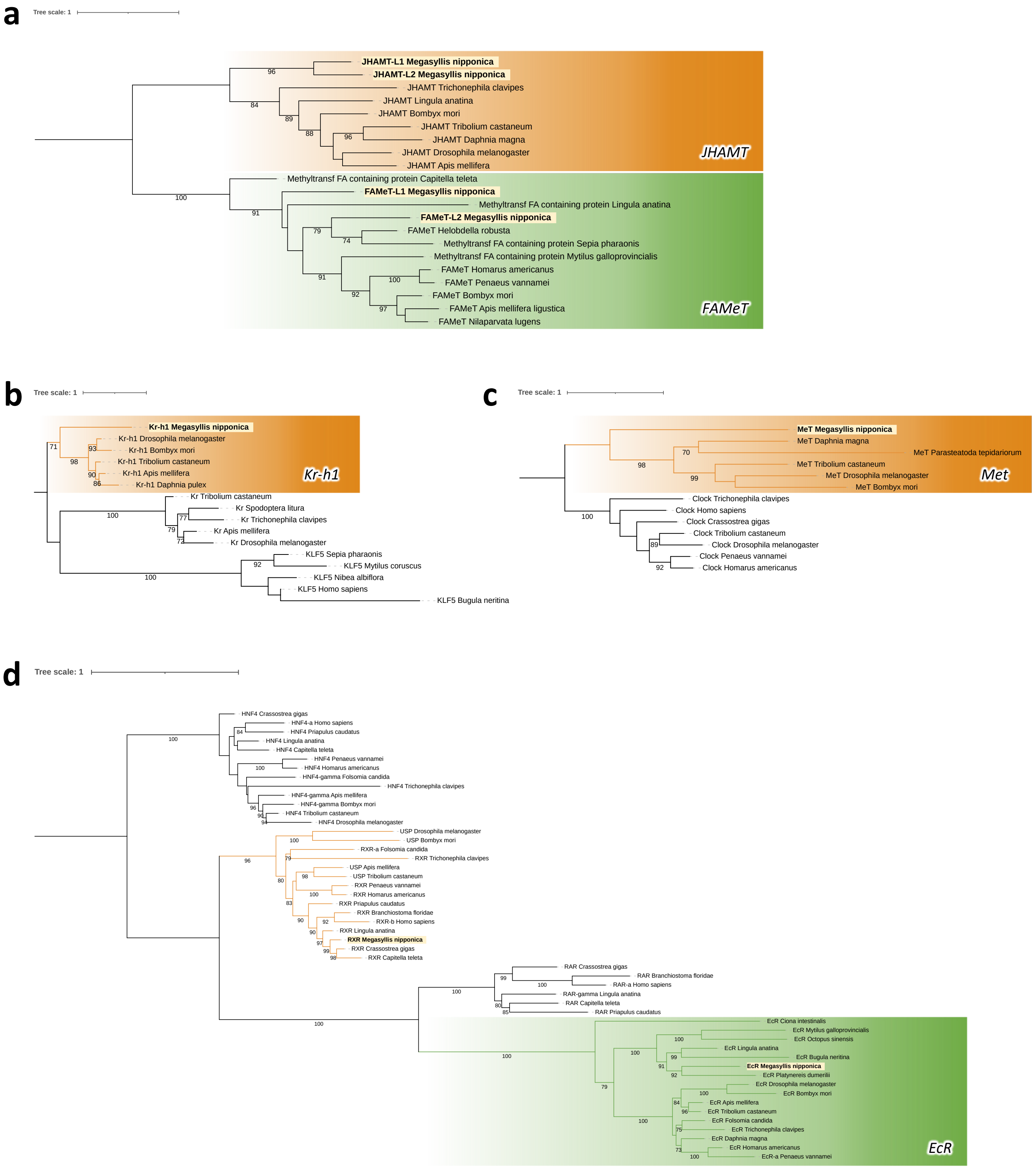

Supplementary Figure 8.

Molecular phylogenetic trees of hormone-related genes. Maximum likelihood phylogenetic analyses of *JHAMT* [*Juvenile hormone acid O-methyltransferase*] and *FAMeT* [*Farnesoic acid methyl transferase*] (**a**), *Kr-h1* [*Krüppel homolog 1*] (**b**), *Met* [*Methoprene-tolerant*] (**c**), and *EcR* [*Ecdysone Receptor*] (**d**). *Kr* [*Krüppel*] and *KLF5* [*Krüppel Like Factor 5*] (**b**), *Clock* (**c**), and *HNF4* [*Hepatocyte Nuclear Factor 4*] (**d**) were used as outgroups. Colored boxes indicate ortholog groups. The sequences were aligned and trimmed to include conserved region. As the best-fitting evolutionary model (**a**) VT+G4, (**b**) DCMut+G4, (**c**) LG+G4, and (**d**) LG+I+G4 were chosen. *Megasylllis nipponica* sequences are highlighted by yellow. Only bootstrap values of at least 70 at nodes that supposedly represent distinct orthology groups are shown.

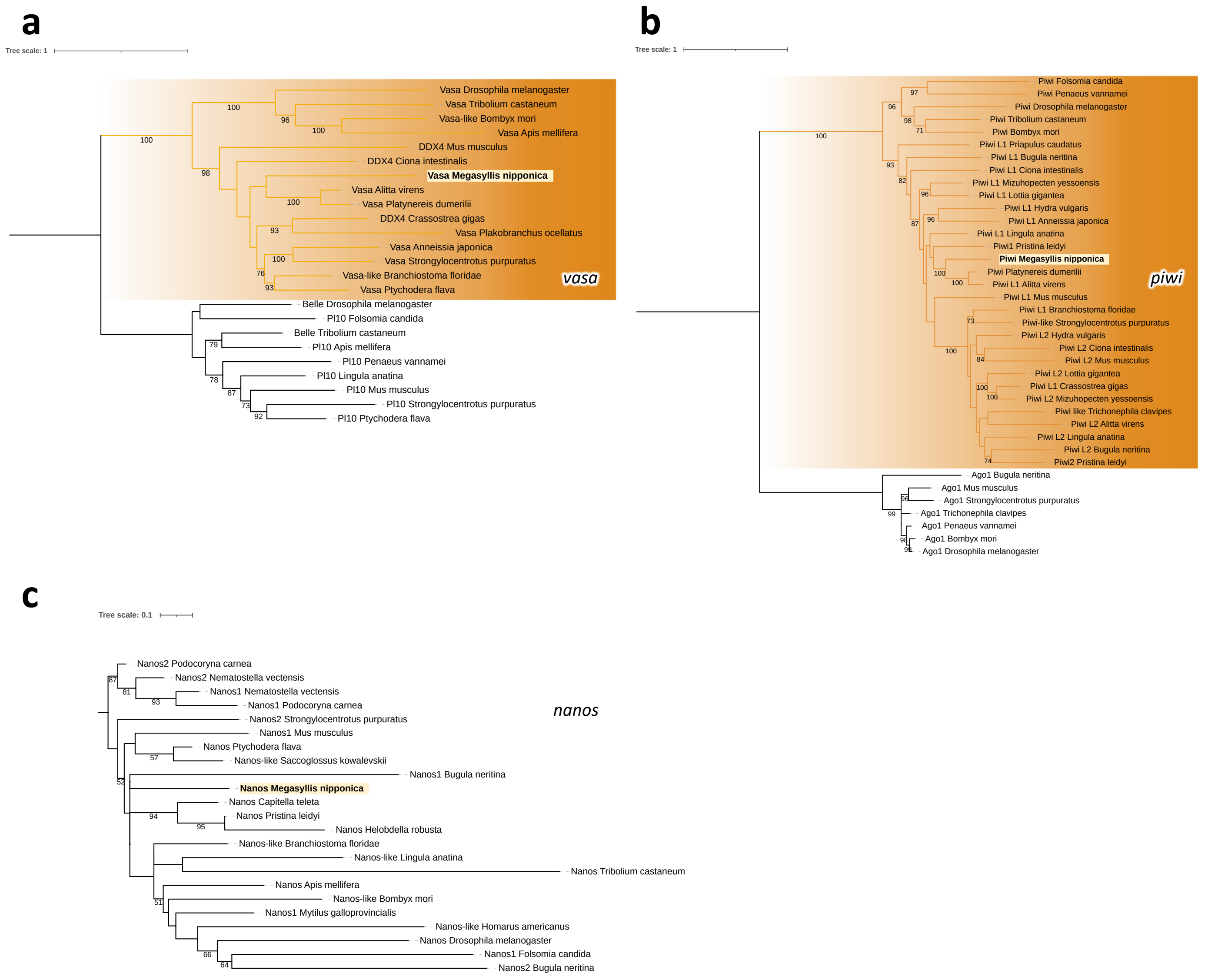

Supplementary Figure 9.

Molecular phylogenetic trees of gonad-development genes. Maximum likelihood phylogenetic analyses of *vasa* (**a**), *piwi* (**b**), and *nanos* (**c**). *Pl10* (**a**) and *Ago1* (**b**) were used as outgroups. Colored boxes indicate ortholog groups. The sequences were aligned and trimmed to include conserved region. As the best-fitting evolutionary model (**a**) LG+F+R5, (**b**) LG+F+I+G4, and (**c**) WAG+G4 were chosen. *Megasylllis nipponica* sequences are highlighted by yellow. Only bootstrap values of at least 70 at nodes for (**a**) and (**b**), and 50 at nodes for (**c**) that supposedly represent distinct orthology groups are shown.
