# Supplementary material for: Morphological, histological and gene-expression analyses on stolonization in the Japanese Green Syllid, Megasyllis nipponica (Annelida, Syllidae)
Source: Sci Rep. 2023 Nov 22;13:19419. doi: 10.1038/s41598-023-46358-8 (PMC10665476; doi:10.1038/s41598-023-46358-8)
Supplement: Supplementary file 2 — Supplementary Information 2. [file 41598_2023_46358_MOESM2_ESM.docx]

**Supplementary Table 1.**

List of COI genes used for the molecular phylogenetic examination of the focal species.

| species name | gene accession number |
| --- | --- |
| *Syllis ferrani* | EF123775.1 |
| *Megasyllis inflata* | JF903776.1 |
| *Megasyllis corruscans* | KM277828.2 |
| *Syllis compacta* | EF123772.1 |
| *Syllis okadai* | EF123783.1 |
| *Syllis ehlersioides* | EF123773.1 |
| *Syllis marugani* | EF123780.1 |
| Megasyllis subantennata | JF903775.1 |
| *Megasyllis niponica*_Mnzr | EF123782.1 |
| *Megasyllis niponica*_Msk_14 | OR381444 |
| *Megasyllis niponica*_Msk_13 | OR381445 |
| *Megasyllis niponica*_Msk_03 | OR381446 |
| *Megasyllis niponica*_Msk_05 | OR381447 |
| *Megasyllis niponica*_Msk_15 | OR381448 |
| *Megasyllis niponica*_Msk_07 | OR381449 |
| *Megasyllis niponica*_Msk_01 | OR381450 |
| *Megasyllis niponica*_Osr_17 | OR381451 |
| *Megasyllis niponica*_Msk_09 | OR381452 |
| *Megasyllis niponica*_Msk_12 | OR381453 |
| *Megasyllis niponica*_Osr_16 | OR381454 |
| *Megasyllis niponica*_Osr_18 | OR381455 |
| *Megasyllis niponica*_Osr_19 | OR381456 |
