# Supplementary material for: Morphological, histological and gene-expression analyses on stolonization in the Japanese Green Syllid, Megasyllis nipponica (Annelida, Syllidae)
Source: Sci Rep. 2023 Nov 22;13:19419. doi: 10.1038/s41598-023-46358-8 (PMC10665476; doi:10.1038/s41598-023-46358-8)
Supplement: Supplementary file 3 — Supplementary Information 3. [file 41598_2023_46358_MOESM3_ESM.docx]

**Supplementary Table 2.**

List of Hox genes used for the orthog searches in *Megasyllis nippponica*.

| **Organism** | **Gene** | **Database** | **Accession no.** |
| --- | --- | --- | --- |
| *Branchiostoma floridae* | Hox1 | GenBank | BAA78620 |
|  | Hox2 |  | BAA78621 |
|  | Hox3 | NCBI | XP_035657468 |
|  | Hox4 | GenBank | BAA78622 |
|  | Hox5 |  | CAA84517 |
|  | Hox6 |  | CAA84518 |
|  | Hox7 |  | CAA84519 |
|  | Hox8 |  | CAA84520 |
|  | Hox9 |  | CAA84521 |
|  | Hox10 |  | CAA84522 |
|  | Hox11 |  | AAF81909 |
|  | Hox12 |  | AAF81903 |
|  | Hox13 |  | AAF81904 |
|  | Hox14 |  | AAF81905 |
|  | Hox15 |  | ACJ74394.1 |
|  | Gsx |  | AAC39015.1 |
|  | Xlox |  | AAC39016.1 |
|  | Cdx |  | AAC39017 |
|  | Evx-a |  | AAK58953.1 |
|  | Evx-b |  | AAK58954.1 |
| *Bugula turrita* | pb | GenBank | AAS77225 |
|  | Hox3 |  | AAS77226 |
|  | Dfd-a |  | AAS77227 |
|  | Dfd-b |  | AAS77228 |
|  | Lox5 |  | AAS77229 |
|  | Post2 |  | AAS77230 |
| *Capitella teleta* | lab | GenBank | ABY67952 |
|  | pb |  | ABY67953 |
|  | Hox3 |  | ABY67954 |
|  | Dfd |  | ABY67955 |
|  | Scr |  | ABY67956 |
|  | Lox5 |  | ABY67957 |
|  | Antp |  | ABY67962 |
|  | Lox4 |  | ABY67958 |
|  | Lox2 |  | ABY67959 |
|  | Post1 |  | ABY67961 |
|  | Post2 |  | ABY67960 |
|  | Gsx |  | AAZ23124.1 |
|  | Cdx |  | AAZ95508 |
|  | Xlox |  | AAZ95509.1 |
|  | Evx |  | ABG82164 |
| *Crassostrea gigas* | Hox1 | ENSEMBL | CGI_10024083 |
|  | Hox2 |  | CGI_10024086 |
|  | Hox3 |  | CGI_10024087 |
|  | Hox4 |  | CGI_10024091 |
|  | Hox7 |  | CGI_10026565 |
|  | Lox2 |  | CGI_10018592 |
| *Drosophila melanogaster* | lab | GenBank | CAB57787 |
|  | pb |  | CAA45271 |
|  | Zen |  | AAF54087.1 |
|  | Zen2 |  | P09090.2 |
|  | Dfd |  | P07548 |
|  | Scr | NCBI | NP_524248 |
|  | ftz |  | NP_477498 |
|  | Antp | GenBank | CAA27417 |
|  | Ubx |  | CAA29194 |
|  | Abd-A |  | P29555 |
|  | Abd-B |  | CAB57859 |
|  | Ind | NCBI | NP_996087.2 |
|  | Cad | GenBank | AAA28409.1 |
|  | Eve | NCBI | NP_523670.2 |
| *Euprymna scolopes* | lab | GenBank | AY330184 |
|  | Hox3 |  | AY330185 |
|  | Scr |  | AAL25807.1 |
|  | Lox5 |  | AY330187 |
|  | Antp |  | AY330188 |
|  | Lox4 |  | AAL25810.1 |
|  | Post1 |  | AY330190 |
|  | Post2 |  | AY330191 |
| *Flaccisaggita enflata* | Hox1 | GenBank | ABS18809.1 |
|  | Hox3 |  | ABS18810.1 |
|  | Hox4 |  | ABS18811.1 |
|  | Hox5 |  | ABS18812.1 |
|  | Hox6 |  | ABS18813.1 |
|  | Hox8 |  | ABS18814.1 |
|  | MedPost |  | ABS18817.1 |
|  | Posta |  | ABS18815.1 |
|  | Postb |  | ABS18816.1 |
| *Gibbula varia* | Hox1 | GenBank | ACX84671.1 |
|  | Hox2 |  | ADJ18233.1 |
|  | Hox3 |  | ADJ18232.1 |
|  | Hox4 |  | ACX84672.1 |
|  | Hox5 |  | ADJ18234.1 |
|  | Lox5 |  | ADJ18235.1 |
|  | Hox7 |  | ADJ18236.1 |
|  | Lox2 |  | ADJ18238.1 |
|  | Lox4 |  | ADJ18237.1 |
|  | Post1 |  | ACX84673.1 |
| *Homo sapiens* | HoxA1 | GenBank | AAB35423.2 |
|  | HoxD1 |  | AAG44444.1 |
|  | HoxA2 | NCBI | NP_006726.1 |
|  | HoxB2 |  | NP_002136.1 |
|  | HoxA3 |  | NP_705895.1 |
|  | HoxB3 | GenBank | AAD10852.1 |
|  | HoxD3 |  | CAA71102.1 |
|  | HoxA4 | NCBI | NP_002132.3 |
|  | HoxB4 | GenBank | AAG45052.1 |
|  | HoxC4 |  | AAG42145.1 |
|  | HoxD4 | NCBI | NP_055436.2 |
|  | HoxA5 | GenBank | CAG47052.1 |
|  | HoxB5 | NCBI | NP_002138.1 |
|  | HoxC5 | GenBank | EAW96748.1 |
|  | HoxA6 | NCBI | NP_076919.1 |
|  | HoxB6 |  | NP_061825.2 |
|  | HoxC6 | GenBank | CAG33235.1 |
|  | HoxA7 |  | CAA06713.1 |
|  | HoxB7 | NCBI | NP_004493.3 |
|  | HoxB8 | GenBank | AAG42143.1 |
|  | HoxC8 |  | AAG42146.1 |
|  | HoxD8 |  | AAG42152.1 |
|  | HoxA9 | NCBI | NP_689952.1 |
|  | HoxB9 | GenBank | AAG42144.1 |
|  | HoxC9 |  | AAG42151.1 |
|  | HoxD9 | NCBI | NP_055028.3 |
|  | HoxC10 |  | NP_059105.2 |
|  | HoxD10 |  | NP_002139.2 |
|  | HoxA11 |  | NP_005514.1 |
|  | HoxC11 |  | NP_055027.1 |
|  | HoxD11 | GenBank | AAF79045.1 |
|  | HoxC12 |  | AAK16717.1 |
|  | HoxD12 |  | AAF79044.1 |
|  | HoxA13 |  | AAC50993.1 |
|  | HoxB13 |  | AAH70233.1 |
|  | HoxC13 |  | AAF73439.1 |
|  | HoxD13 |  | AAC51635.1 |
|  | Gsx1 | NCBI | NP_663632.1 |
|  | Gsx2 |  | NP_573574.1 |
|  | Pdx1 |  | NP_000200.1 |
|  | Cdx-1 |  | NP_001795.2 |
|  | Cdx-2 |  | NP_001256.3 |
|  | Cdx-4 |  | NP_005184.1 |
|  | Evx-1 |  | NP_001291448.1 |
|  | Evx-2 |  | NP_001073927.1 |
| *Lingula anatina* | lab | ENSEMBL | g10891 |
|  | pb |  | g10890 |
|  | Hox3 |  | g10889 |
|  | Dfd |  | g10888 |
|  | Scr |  | g10887 |
|  | Lox5 |  | g10886 |
|  | Antp |  | g10892 |
|  | Post1 |  | g12396 |
|  | Post2 |  | g12399 |
| ***Megasyllis nipponica*** | Hox1 | Hayashi et al. 2022 | DN26841_c4_g1 |
|  | Hox2 |  | DN19818_c0_g3 |
|  | Hox3 |  | DN21080_c1_g2 |
|  | Hox4 |  | DN30959_c0_g1 |
|  | Hox5 |  | DN30312_c2_g2 |
|  | Hox7 |  | DN27734_c0_g1 |
|  | Lox5 |  | DN23954_c2_g2 |
|  | Lox4 |  | DN31520_c0_g1 |
|  | Lox2 |  | DN24063_c0_g1 |
|  | Post2 |  | DN29598_c3_g1 |
| *Micrura alaskensis* | lab | GenBank | KP762174 |
|  | pb |  | KP762176 |
|  | Hox3 |  | KP762173 |
|  | Dfd |  | KP762180 |
|  | Scr |  | KP762177 |
|  | Lox5 |  | KP762179 |
|  | Antp |  | KP762171 |
|  | Lox4 |  | AKE07581.1 |
|  | Post2 |  | KP762178 |
| *Platynereis dumerilli* | Hox1 | GenBank | AFJ91921.1 |
|  | Hox2 |  | AFJ91922.1 |
|  | Hox3 |  | AFJ91923.1 |
|  | Hox4 |  | AFJ91924.1 |
|  | Hox5 |  | ATG29892.1 |
|  | Hox7 |  | ATG29893.1 |
|  | Lox5 |  | AFJ91925.1 |
|  | Lox2 |  | AFJ91926.1 |
|  | Post2 |  | AFJ91927.1 |
|  | Post1 |  | AFJ91928.1 |
| *Priapulus caudatus* | lab | GenBank | AAD40640.1 |
|  | pb |  | AAD40641.1 |
|  | Hox3 |  | AAD40642.1 |
|  | Dfd |  | AAD40643.1 |
|  | Ubx |  | AAD40647.1 |
|  | Abd-B |  | AAD40649.1 |
| *Ptychodera flava* | Hox1 | GenBank | AAR07634.1 |
|  | Hox4 |  | AAR07635.1 |
|  | Hox5 |  | AAR07636.1 |
|  | Hox6 |  | AAR07637.1 |
|  | Hox9/10 |  | AAR07638.1 |
|  | Hox11/13a |  | AAR07639.1 |
|  | Hox11/13b |  | AAR07640.1 |
|  | Hox11/13c |  | AAR07641.1 |
|  | Xlox1 |  | AAR07643.1 |
|  | Xlox2 |  | AAR07644.1 |
| *Saccoglossus kowalevskii* | Gsx | Uniprot | A0A0U2UDE9 |
|  | Cdx | NCBI | NP_001158415.1 |
|  | Evx |  | NP_001164694.1 |
| *Tribolium castaneum* | lab | GenBank | EEZ99257.1 |
|  | Mxp | NCBI | NP_001107807.1 |
|  | Zen1 |  | NP_001036813 |
|  | Zen2 | GenBank | AAK16425.1 |
|  | Dfd |  | AAK16423.1 |
|  | Cx | NCBI | NP_001034523.1 |
|  | ftz | GenBank | AAK16421.1 |
|  | Ptl | NCBI | NP_001034505.1 |
|  | Utx | GenBank | EEZ99249.1 |
|  | Abd-A |  | EEZ99248.1 |
|  | Abd-B |  | EEZ99247.1 |
|  | Ind |  | AAW21974.1 |
|  | Cad-1 | NCBI | NP_001034498.1 |
|  | Cad-2 |  | XP_008191732.1 |
|  | Eve |  | NP_001034538.1 |
