# Supplementary material for: Morphological, histological and gene-expression analyses on stolonization in the Japanese Green Syllid, Megasyllis nipponica (Annelida, Syllidae)
Source: Sci Rep. 2023 Nov 22;13:19419. doi: 10.1038/s41598-023-46358-8 (PMC10665476; doi:10.1038/s41598-023-46358-8)
Supplement: Supplementary file 4 — Supplementary Information 4. [file 41598_2023_46358_MOESM4_ESM.docx]

**Supplementary Table 3.**

List of head-identification genes used for the orthog searches in *Megasyllis nippponica*.

| **Organism** | **Gene** | **Database** | **Accession no.** |
| --- | --- | --- | --- |
| *Branchiostoma floridae* | otx | GenBank | AAC00193.1 |
|  | pax6 |  | CAA11368.1 |
|  | pax2-a |  | AAC12733.1 |
| *Crassostrea gigas* | otx5-b | NCBI | XP_011415946.1 |
|  | orthopedia |  | XP_011436433.1 |
| *Drosophila melanogaster* | optix-a | NCBI | NP_524695.2 |
|  | six4-a |  | NP_649256.1 |
|  | vnd-b |  | NP_001036253.1 |
|  | orthopedia-l |  | NP_001356966.1 |
|  | eyeless-a |  | NP_524628.2 |
|  | shaven-d |  | NP_001014701.1 |
| *Euprymna scolopes* | pax6 | GenBank | AHA91756.1 |
| *Homo sapiens* | six3 | NCBI | NP_005404.1 |
|  | six4 |  | NP_059116.3 |
|  | otx1 |  | NP_001186699.1 |
|  | orthopedia |  | NP_115485.1 |
|  | pax6-b |  | NP_001355823.1 |
|  | pax2-a |  | NP_003978.3 |
|  | Nkx2.1-1 |  | NP_001073136.1 |
|  | Nkx2.2 |  | NP_002500.1 |
| *Lingula anatina* | otx2-b | NCBI | XP_013379092.1 |
|  | orthopedia |  | XP_013394470.1 |
|  | pax6 |  | XP_023930086.1 |
|  | pax2-b |  | XP_013394327.1 |
| ***Megasyllis nipponica*** | six3 | Hayashi et al. 2022 | DN32351_c2_g3 |
|  | otx |  | DN22463_c0_g1 |
|  | pax6 |  | DN26882_c0_g1 |
|  | nk2.1 |  | DN27127_c0_g1 |
| *Micrura alaskensis* | six3/6 | GenBank | AKE07578.1 |
| *Platynereis dumerilli* | six3 | GenBank | CAR66435.1 |
|  | otx |  | CAC19028.1 |
|  | orthopedia |  | ABR68849.1 |
|  | pax6 |  | CAJ40659.1 |
|  | nk2.1 |  | CAJ38809.1 |
|  | nk2.2 |  | ABO93209.1 |
| *Priapulus caudatus* | orthodenticle | GenBank | AFY12008.1 |
| *Ptychodera flava* | six3 | GenBank | AJS19019.1 |
|  | otx |  | BAA89013.1 |
|  | nk2.1 |  | AAM93268.1 |
| *Saccoglossus kowalevskii* | six3 | NCBI | NP_001158378.1 |
|  | orthodenticle |  | NP_001158360.1 |
|  | orthopedia |  | NP_001158374.1 |
|  | pax6 |  | NP_001158383.1 |
|  | pax2-b |  | XP_006813069.1 |
|  | nk2.1 |  | NP_001158382.1 |
|  | nk2.2 |  | NP_001158404.1 |
| *Tribolium castaneum* | optix-a | NCBI | NP_001106938.1 |
|  | six4 | GenBank | CBL87028.1 |
|  | orthodenticle | NCBI | NP_001034513.1 |
|  | vnd |  | XP_967738.2 |
|  | orthopedia |  | NP_001163995.1 |
|  | eyeless |  | NP_001103907.1 |
|  | shaven | GenBank | KYB28704.1 |
