# Supplementary material for: Morphological, histological and gene-expression analyses on stolonization in the Japanese Green Syllid, Megasyllis nipponica (Annelida, Syllidae)
Source: Sci Rep. 2023 Nov 22;13:19419. doi: 10.1038/s41598-023-46358-8 (PMC10665476; doi:10.1038/s41598-023-46358-8)
Supplement: Supplementary file 5 — Supplementary Information 5. [file 41598_2023_46358_MOESM5_ESM.docx]

**Supplementary Table 4.**

List of hormone-related genes used for the orthog searches in *Megasyllis nippponica*.

| **Organism** | **Gene** | **Database** | **Accession no.** |
| --- | --- | --- | --- |
| *Apis mellifera* | JHAMT | NCBI | NP_001314896.1 |
|  | Kr-h1 |  | NP_001229399.1 |
|  | Kr |  | NP_001239353.1 |
|  | EcR |  | NP_001091685.2 |
|  | USP |  | NP_001011634.1 |
|  | HNF4-gamma |  | XP_026298547.1 |
| *Apis mellifera ligustica* | FAMeT | GenBank | AIE12450.1 |
| *Bombyx mori* | FAMeT | GenBank | AFR54361.1 |
|  | JHAMT | NCBI | XP_037870195.1 |
|  | Kr-h1 |  | NP_001171332.1 |
|  | EcR |  | NP_001037331.2 |
|  | USP |  | NP_001037470.1 |
|  | HNF4-gamma |  | NP_001166834.1 |
|  | MeT |  | NP_001108458.1 |
| *Branchiostoma floridae* | RAR | GenBank | AAM46149.1 |
|  | RXR |  | AAM46151.1 |
| *Bugula neritina* | KLF5 | GenBank | KAF6040058.1 |
|  | EcR |  | AWH55630.1 |
| *Capitella teleta* | Methyltransf_FA | GenBank | ELT97145.1 |
|  | RAR |  | ELU07684.1 |
|  | RXR |  | ELT93409.1 |
|  | HNF4 |  | ELT88964.1 |
| *Ciona intestinalis* | EcR | NCBI | XP_026695913.1 |
| *Crassostrea gigas* | RAR | GenBank | ARM65370.1 |
|  | RXR | NCBI | XP_011434500.1 |
|  | HNF4 |  | XP_034337695.1 |
|  | Clock | GenBank | AQM57601.1 |
| *Daphnia magna* | EcR | GenBank | BAF49030.1 |
|  | MeT |  | BAM83855.1 |
|  | JHAMT |  | BCF86812.1 |
| *Daphnia pulex* | Kr-h1 | GenBank | BBB03674.1 |
| *Drosophila melanogaster* | JHAMT | NCBI | NP_001285980.1 |
|  | Kr-h1 |  | NP_477467.1 |
|  | Kr |  | NP_523867.1 |
|  | EcR |  | NP_001163061.1 |
|  | USP |  | NP_001259168.1 |
|  | HNF4 |  | NP_001285758.1 |
|  | MeT |  | NP_001285132.1 |
|  | Clock |  | NP_996021.2 |
| *Folsomia candida* | EcR | GenBank | OXA57923.1 |
|  | RXR-a | NCBI | XP_035704779.1 |
|  | HNF4-gamma |  | XP_035709469.1 |
| *Helobdella robusta* | FAMeT | NCBI | XP_009028978.1 |
| *Homarus americanus* | FAMeT | GenBank | AAA67081.1 |
|  | EcR |  | AEA29831.1 |
|  | RXR |  | AGI15961.1 |
|  | HNF4 | NCBI | XP_042234157.1 |
|  | Clock | GenBank | AWC08577.1 |
| *Homo sapiens* | KLF5 | GenBank | AER93391.1 |
|  | RAR-a | NCBI | NP_001138773.1 |
|  | RXR-b |  | NP_001278918.1 |
|  | HNF4-a |  | XP_005260464.1 |
|  | Clock | GenBank | AAB83969.1 |
| *Lingula anatina* | Methyltransf_FA | NCBI | XP_023930787.1 |
|  | JHAMT |  | XP_013383203.1 |
|  | EcR |  | XP_013421724.1 |
|  | RAR-gamma |  | XP_013406624.1 |
|  | RXR |  | XP_013412685.1 |
|  | HNF4 |  | XP_013405260.1 |
| *Litopenaeus vannamei* | FAMeT | GenBank | AAZ22180.1 |
|  | EcR-a |  | AGS94405.1 |
|  | RXR |  | ROT73451.1 |
|  | HNF4 |  | ROT83509.1 |
|  | Clock |  | ROT60838.1 |
| ***Megasyllis nipponica*** | FAMeT-L1 | Hayashi et al. 2022 | DN32805_c0_g1 |
|  | FAMeT-L2 |  | DN19499_c0_g1 |
|  | JHAMT-L1 |  | DN30141_c1_g1 |
|  | JHAMT-L2 |  | DN33052_c0_g1 |
|  | Kr-h1 |  | DN26344_c0_g1 |
|  | EcR |  | DN33364_c1_g1 |
|  | RXR |  | DN27563_c1_g1 |
|  | MeT |  | DN25616_c0_g1 |
| *Mytilus coruscus* | KLF5 | GenBank | CAC5381853.1 |
| *Mytilus galloprovincialis* | Methyltransf_FA | GenBank | VDI67056.1 |
|  | EcR |  | VDI41127.1 |
| *Nibea albiflora* | KLF5 | GenBank | KAG8000538.1 |
| *Nilaparvata lugens* | FAMeT | GenBank | ACL26692.1 |
| *Octopus sinensis* | EcR | NCBI | XP_029636349.1 |
| *Parasteatoda tepidariorum* | MeT | GenBank | QUS52446.1 |
| *Platynereis dumerilii* | EcR | GenBank | AWH55629.1 |
| *Priapulus caudatus* | RAR | GenBank | QFQ33539.1 |
|  | RXR |  | QFQ33540.1 |
|  | HNF4 |  | AKU77016.1 |
| *Sepia pharaonis* | Methyltransf_FA | GenBank | CAE1263810.1 |
|  | KLF5 |  | CAE1283970.1 |
| *Spodoptera litura* | Kr | GenBank | AUO38742.1 |
| *Tribolium castaneum* | JHAMT | NCBI | NP_001120783.1 |
|  | Kr-h1 |  | NP_001129235.1 |
|  | Kr |  | NP_001034527.2 |
|  | EcR |  | NP_001135390.1 |
|  | USP | GenBank | EFA04649.1 |
|  | HNF4 |  | KYB26570.1 |
|  | MeT | NCBI | NP_001092812.1 |
|  | Clock | GenBank | EFA01240.2 |
| *Trichonephila clavipes* | JHAMT | GenBank | PRD35481.1 |
|  | Kr-h1 |  | PRD19382.1 |
|  | Kr |  | PRD32801.1 |
|  | EcR |  | GFX35598.1 |
|  | RXR |  | PRD28408.1 |
|  | HNF4 |  | GFX93709.1 |
|  | Clock |  | GFU38674.1 |
