# Supplementary material for: Morphological, histological and gene-expression analyses on stolonization in the Japanese Green Syllid, Megasyllis nipponica (Annelida, Syllidae)
Source: Sci Rep. 2023 Nov 22;13:19419. doi: 10.1038/s41598-023-46358-8 (PMC10665476; doi:10.1038/s41598-023-46358-8)
Supplement: Supplementary file 6 — Supplementary Information 6. [file 41598_2023_46358_MOESM6_ESM.docx]

**Supplementary Table 5.**

List of gonad-development genes used for the orthog searches in *Megasyllis nippponica*.

| **Organism** | **Gene** | **Database** | **Accession no.** |
| --- | --- | --- | --- |
| *Alitta virens* | Vasa | GenBank | AJW77403.1 |
|  | Piwi L1 |  | AJW77405.1 |
|  | Piwi L2 |  | AJW77406.1 |
| *Anneissia japonica* | vasa | GenBank | BAJ07808.1 |
|  | Piwi L1 | NCBI | XP_033126803.1 |
| *Apis mellifera* | Vasa | NCBI | NP_001035345.1 |
|  | Pl10 |  | XP_391829.3 |
|  | Nanos |  | NP_001035321.1 |
| *Bombyx mori* | Vasa-like | NCBI | NP_001037347.1 |
|  | Piwi | GenBank | BAF98574.1 |
|  | Ago1 | NCBI | NP_001095931.1 |
|  | Nanos-like |  | NP_001098702.1 |
| *Branchiostoma floridae* | Vasa-like | GenBank | ADM26640.1 |
|  | Piwi L1 | NCBI | XP_035670589.1 |
|  | Nanos-like | GenBank | ADM26639.1 |
| *Bugula neritina* | Piwi L1 | GenBank | KAF6020128.1 |
|  | Piwi L2 |  | KAF6036082.1 |
|  | Ago1 |  | KAF6035891.1 |
|  | Nanos1 |  | KAF6040065.1 |
|  | Nanos2 |  | KAF6037289.1 |
| *Capitella teleta* | Nanos | GenBank | DAA06318.1 |
| *Ciona intestinalis* | DDX4 | NCBI | NP_001027593.1 |
|  | Piwi L1 |  | XP_026692676.1 |
|  | Piwi L2 |  | XP_002130577.2 |
| *Crassostrea gigas* | DDX4 | NCBI | NP_001292258.1 |
|  | Piwi L1 |  | XP_034311101.1 |
| *Drosophila melanogaster* | Vasa | NCBI | NP_723899.1 |
|  | Belle |  | NP_536783.1 |
|  | Piwi |  | NP_476875.1 |
|  | Ago1 |  | NP_523734.1 |
|  | Nanos |  | NP_476658.1 |
| *Folsomia candida* | Pl10 | NCBI | XP_035703716.1 |
|  | Piwi |  | XP_021961202.2 |
|  | Nanos1 | GenBank | OXA45488.1 |
| *Helobdella robusta* | Nanos | GenBank | AAB63111.1 |
| *Homarus americanus* | Nanos-like | GenBank | KAG7170670.1 |
| *Hydra vulgaris* | Piwi L1 | NCBI | NP_001274302.1 |
|  | Piwi L2 |  | NP_001296620.1 |
| *Lingula anatina* | Belle | NCBI | XP_013405265.1 |
|  | Piwi L1 |  | XP_013405373.1 |
|  | Piwi L2 |  | XP_013382739.1 |
|  | Nanos-like |  | XP_013398541.1 |
| *Litopenaeus vannamei* | Pl10 | GenBank | ROT75338.1 |
|  | Piwi |  | ROT71554.1 |
|  | Ago1 |  | ROT79503.1 |
| *Lottia gigantea* | Piwi L1 | NCBI | XP_009064630.1 |
|  | Piwi L2 |  | XP_009065055.1 |
| *Mizuhopecten yessoensis* | Piwi L1 | GenBank | OWF41593.1 |
|  | Piwi L2 |  | OWF42401.1 |
| ***Megasyllis nipponica*** | Vasa | Hayashi et al. 2022 | DN29513_c0_g2 |
|  | Piwi |  | DN29626_c7_g4 |
|  | Nanos |  | DN30515_c2_g4 |
| *Mus musculus* | DDX4 | NCBI | NP_001139357.1 |
|  | Pl10 |  | NP_149068.1 |
|  | Piwi L1 |  | NP_067286.1 |
|  | Piwi L2 |  | NP_001351250.1 |
|  | Ago1 |  | NP_700452.2 |
|  | Nanos1 | GenBank | BAC76003.1 |
| *Mytilus galloprovincialis* | Nanos1 | GenBank | VDI80212.1 |
| *Nematostella vectensis* | Nanos1 | GenBank | AAW29070.1 |
|  | Nanos2 |  | AAW29071.1 |
| *Plakobranchus ocellatus* | Vasa | GenBank | GFO03223.1 |
| *Platynereis dumerilii* | Vasa | GenBank | CAJ38803.1 |
|  | Piwi |  | CAJ28986.1 |
| *Podocoryna carnea* | Nanos1 | GenBank | AAU11513.1 |
|  | Nanos2 |  | AAU11514.1 |
| *Priapulus caudatus* | Piwi L1 | NCBI | XP_014672865.1 |
| *Pristina leidyi* | Piwi1 | GenBank | AIM52094.1 |
|  | Piwi2 |  | AIM52095.1 |
|  | Nanos |  | ADE44350.1 |
| *Ptychodera flava* | Vasa | GenBank | QOW95195.1 |
|  | Pl10 |  | QOW95196.1 |
|  | Nanos |  | QOW95197.1 |
| *Saccoglossus kowalevskii* | Nanos-like | NCBI | NP_001161595.1 |
| *Strongylocentrotus purpuratus* | Vasa | NCBI | NP_001139665.1 |
|  | Pl10 |  | XP_030837315.1 |
|  | Piwi L1 |  | XP_030835319.1 |
|  | Ago1 |  | NP_001124190.1 |
|  | Nanos2 |  | NP_001073023.1 |
| *Tribolium castaneum* | Vasa | NCBI | NP_001034520.2 |
|  | Belle |  | NP_001153721.1 |
|  | Piwi |  | XP_015837420.1 |
|  | Nanos | GenBank | EEZ99428.2 |
| *Trichonephila clavipes* | Piwi-like | GenBank | PRD22810.1 |
|  | Ago1 |  | PRD26587.1 |
