# Supplementary material for: Morphological, histological and gene-expression analyses on stolonization in the Japanese Green Syllid, Megasyllis nipponica (Annelida, Syllidae)
Source: Sci Rep. 2023 Nov 22;13:19419. doi: 10.1038/s41598-023-46358-8 (PMC10665476; doi:10.1038/s41598-023-46358-8)
Supplement: Supplementary file 7 — Supplementary Information 7. [file 41598_2023_46358_MOESM7_ESM.docx]

**Supplementary Table 6.**

List of primer sets used for qRT-PCR.

| **Gene** |  | **Seaquences** |
| --- | --- | --- |
| *Rp49* | F: | AAGCAAAACTGGAGGAAGCC |
|  | R: | TGTTCGGCATCTTGTACTGC |
| *six3* | F: | TGCGTCATTTGCACCTTTGG |
|  | R: | TAGAAATAGTCCAGCGGCAGAC |
| *otx* | F: | AGGTCGCGCTCAAAATCAAC |
|  | R: | TGCACTTAGCTCTGCGATTC |
| *pax6* | F: | TTCGTCAACGGGCGGCCTTTAC |
|  | R: | CAGCCGTTGGAGACCTGGAGGA |
| *nk2.1* | F: | CCCAACACAGGTGAAAATCTGG |
|  | R: | TGCTGCGAGTCCATCTTTTC |
| *Hox1* | F: | ACCAGCACAAAAAGCGGAAC |
|  | R: | AATTGGTCCGTCCCATGTTG |
| *Hox2* | F: | TGATCAGGATGTGGGGAAAGTG |
|  | R: | TTTTGCTGCTGGTGGTGTTG |
| *Hox4* | F: | AACGGCATGACACAGTGTTC |
|  | R: | ATTGCTGCTGGTTTGTTGGG |
| *Hox5* | F: | GGTGGAGGTGGCGGTGAAAGTG |
|  | R: | GACCCGGCGCTGTTGTAGTTCC |
| *Lox5* | F: | TGCAGCTGAATTTGGCTACG |
|  | R: | GACCAAGGGCATGTGCTATTTC |
| *Hox7* | F: | ACAAACACCGCAACAGCATC |
|  | R: | TTGTTGTGGCTGGAGCTAGG |
| *Lox4* | F: | AATAGAGATCGCGCATGCAC |
|  | R: | TGCTGGCGCTCTTTCTTTAG |
| *Lox2* | F: | TTGATCTGCCGCATTCCTTG |
|  | R: | TGTTGTTGTGGTGGTGTTGG |
| *Post2* | F: | ACGCCTCCTCCTTCACAGGGTC |
|  | R: | CAACTGGACCTGCTGGCACTGG |
| *FAMeT L1* | F: | TGGTGTTCTTGGTTCAAGCG |
|  | R: | TTGCTGTTTTCGCTCATGCC |
| *FAMeT L2* | F: | TGGAATCATGCAGTGACGTC |
|  | R: | AGGCGAGTTATGGTCGTTTG |
| *JHAMT L1* | F: | TGGACACAAAACAGGCTTGC |
|  | R: | CTGCAATTTGCTGGCAAGTC |
| *JHAMT L2* | F: | TGCGTTGGCTGAGAAATTCC |
|  | R: | TTCGCTGTTGCTATGGCTTC |
| *MeT* | F: | TGACGCTGAAATGGCAAACC |
|  | R: | AGCTGCATCGTACTGTGTTG |
| *Kr-h1* | F: | ATAGCCAAAACCACCATGCG |
|  | R: | TTGCCGCAAATGTCACACTC |
| *EcR* | F: | ACCATTGTGTTTGCCGATGG |
|  | R: | ATTGTCGACGGCCAGTTTAC |
| *vasa* | F: | TATGGCCTGTGCTCAAACAG |
|  | R: | AATCACAATGGCAGCTGGTG |
| *piwi* | F: | ACAGCCCAACAACAATGCAG |
|  | R: | TGTTGCTTCACAAGGACAGC |
| *nanos* | F: | AATGGTGATGTTGCGCACAC |
|  | R: | ATCTTTGGCAGGGCTGTTTC |
